# Chronic Exposure to Type-I IFN under Lymphopenic Conditions Alters CD4 T Cell Homeostasis

**DOI:** 10.1371/journal.ppat.1003976

**Published:** 2014-03-06

**Authors:** Cecile Le Saout, Rebecca B. Hasley, Hiromi Imamichi, Lueng Tcheung, Zonghui Hu, Megan A. Luckey, Jung-Hyun Park, Scott K. Durum, Mindy Smith, Adam W. Rupert, Michael C. Sneller, H. Clifford Lane, Marta Catalfamo

**Affiliations:** 1 CMRS/Laboratory of Immunoregulation, NIAID, NIH, Bethesda, Maryland, United States of America; 2 Biostatistics Research Branch, DCR, NIAID, NIH, Bethesda, Maryland, United States of America; 3 Experimental Immunology Branch, CCR, NCI, NIH, Bethesda, Maryland, United States of America; 4 Laboratory of Immunoregulation, CCR, NCI, NIH, Frederick, Maryland, United States of America; 5 AIDS Monitoring Labs. Leidos Biomedical Research, Inc, Frederick, Maryland, United States of America; Emory University, United States of America

## Abstract

HIV infection and the associated chronic immune activation alter T cell homeostasis leading to CD4 T cell depletion and CD8 T cell expansion. The mechanisms behind these outcomes are not totally defined and only partially explained by the direct cytopathic effect of the virus. In this manuscript, we addressed the impact of lymphopenia and chronic exposure to IFN-α on T cell homeostasis. In a lymphopenic murine model, this interaction led to decreased CD4 counts and CD8 T cell expansion in association with an increase in the Signal Transducer and Activator of Transcription 1 (STAT1) levels resulting in enhanced CD4 T cell responsiveness to IFN-α. Thus, in the setting of HIV infection, chronic stimulation of this pathway could be detrimental for CD4 T cell homeostasis.

## Author Summary

While the acute CD4 depletion observed in the initial phase of HIV infection is likely due to direct cytopathic effects of the virus, the mechanism/s underlying the steady decline of the CD4 T cell pool during the chronic phase of infection are unclear and are felt to be associated with “immune activation.” We hypothesized that the combination of two distinct forces: homeostatic (CD4 T cell depletion) and inflammatory (HIV-driven IFN-α), lead to a form of T cell activation that results in a decline in the CD4 T cell pool and an increase in the CD8 T cells. IL-7 and lymphopenia enhanced CD4 T cell responsiveness to IFN-α by modulating expression of the Signal Transducers and Activators of Transcription 1, 2 and 3. In a murine model, CD4 T cell depletion and CD8 T cell expansion were observed in a lymphopenic host chronically treated with IFN-α. These findings suggest that a synergistic interaction between lymphopenia and IFN-α may play a role in the pathogenesis of HIV infection. The analysis of this pathway may contribute to the development of new strategies to reverse the dysregulation of the T cell pools seen in patients with HIV infection.

## Introduction

Homeostatic forces regulate the number and survival of T cell clones throughout life, allowing only a limited degree of non-antigen driven expansion for each individual cell in order to preserve the diversity of the T cell repertoire [1]. This is achieved by a balance between signals that mediate survival and proliferation, which are regulated by homeostatic cytokines such as IL-7 and IL-15. Through homeostatic mechanisms, the size of the T cell pool remains relatively constant despite the expansion of T cell clones during antigen-specific responses. In an immune competent host, homeostatic proliferation is controlled by the limited availability of homeostatic cytokines. However, under lymphopenic conditions, a robust homeostatic proliferation occurs leading to polyclonal T cell expansion and accumulation of cells with a highly activated memory phenotype [1]. This is observed in certain human clinical conditions such as bone marrow transplants and HIV infection, where an increased availability of IL-7 is detected in the serum of the patients [2]–[4]. During HIV infection, in addition to HIV-specific immune responses, there is a generalized immune activation that alters the homeostasis of the CD4 and CD8 T cell pools leading to CD4 T cell depletion and CD8 T cell expansion. The mechanisms behind these extreme outcomes are not totally understood. The direct cytopathic effects of HIV do not appear adequate to explain this dichotomy. HIV-induced CD4 T cell depletion triggers a homeostatic response that occurs in an inflammatory environment rich in Type-I IFNs and driven by HIV replication. Both lymphopenia and viral load contribute to the immune activation observed in the CD4 T cell pool. In contrast, the expansion and activation of the CD8 T cell pool is tightly correlated with levels of HIV replication and does not appear influenced by homeostatic forces [5]–[9]. The Type-I IFN activity associated with HIV infection is reflected by increased mRNA transcripts of genes such as OAS1, ISG15, IFNAR1 and STAT1 in both CD4 and CD8 T cells [10]–[12].

Type-I IFN signals through a receptor consisting of two subunits (IFNAR1 and IFNAR2) complexed with JAK1 and TYK2. Activation of these tyrosine kinases leads to the phosphorylation of Signal Transducers and Activators of Transcription 1, 2, 3 and 5 (STAT1, -2, -3 and -5) [13]. While Type-I IFNs are critical for antiviral immunity, in the setting of chronic HIV/SIV infection, chronic exposure has been suggested to play a role in the pathogenesis of the infection, a distinguishing feature of pathogenic from non-pathogenic SIV infection [10], [14], [15]. To understand the mechanisms by which HIV infection alters the homeostasis of CD4 and CD8 T cell pools, we hypothesized that lymphopenia and the chronic exposure to IFN-α may both play a role in this dysregulation. In the present manuscript, we show that IL-7 *in vitro* or lymphopenia *in vivo* can upregulate the total levels of STAT1, -2 and -3, rendering CD4 T cells more sensitive to IFN-α. Levels of total STAT1 (t-STAT1) were associated with the degree of lymphopenia and IL-7 serum levels in HIV-infected patients. In a murine model, lymphopenia and chronic treatment with IFN-α led to diminished survival of CD4 T cells and an expansion of CD8 T cells, thus recapitulating the alterations of the homeostasis of these pools observed in patients with HIV infection. In addition, these data provide new evidence that IL-7 *in vitro* can enhance Type-I IFN responses by modulating the levels of the STATs. This effect could enhance T cell effector differentiation and be advantageous in host defense against pathogens. However, chronic stimulation of this pathway in the setting of lymphopenia and uncontrolled HIV viral replication could be detrimental for CD4 T cell homeostasis and may contribute to the aberrant immune activation and eventual CD4 T cell depletion observed in these patients. The analysis of these pathways can contribute to the development of new strategies to reverse the dysregulation in the T cell pools seen in patients with HIV infection.

## Results

### Total levels of STAT1 are increased in T cells from HIV-infected individuals with high viral loads

Previous studies have demonstrated that *in vivo* CD4 T cell proliferation in patients with HIV infection is correlated with both CD4 T cell counts and HIV-RNA levels [11], [12]. In addition, CD4 T cells from viremic HIV-infected patients showed an enhanced response to *in vitro* stimulation with IFN-α measured by phosphorylation of STAT1 [12]. Because of the potential implication of chronic stimulation of this pathway in CD4 T cell homeostasis, we investigated the mechanisms involved in this process. We hypothesized that this enhanced response could be due to increases in the levels of proteins involved in Type-I IFN signaling pathway, such as IFNAR1 and STATs.

**Figure 1:**
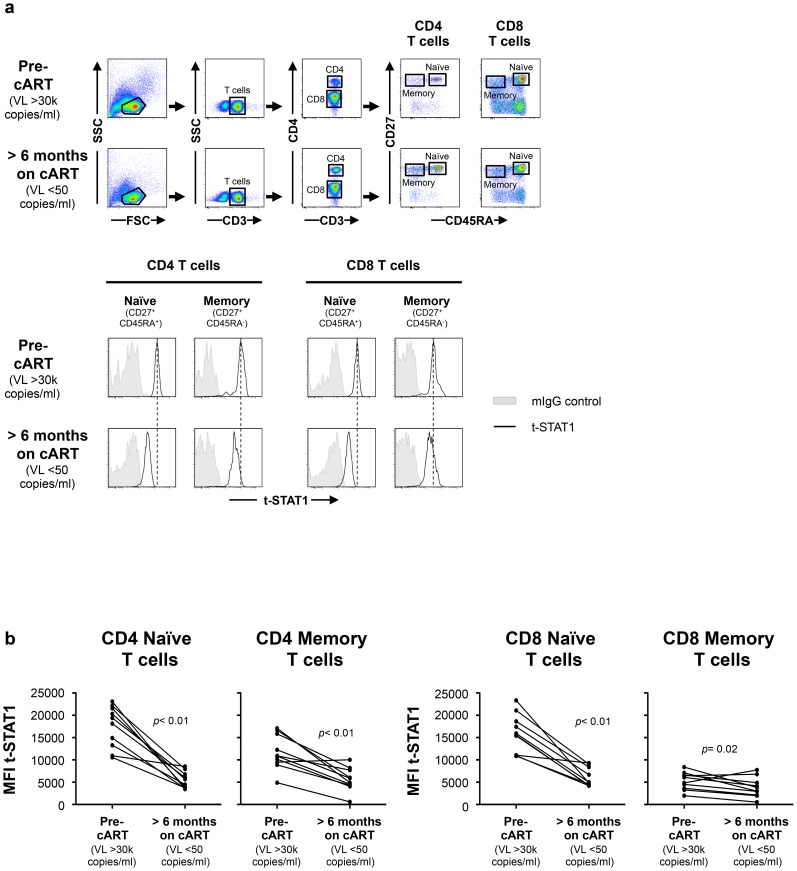
t-STAT1 in CD4 and CD8 T cell subsets from HIV-infected patients. PBMCs from a longitudinal cohort of HIV-infected individuals (n = 10) were analyzed by flow cytometry before and after suppressing viremia to <50 copies/ml with cART for intracellular expression of t-STAT1. (**a**) Gating strategy to assess t-STAT1 expression in T cell subsets, using CD27 and CD45RA as markers of naïve (CD45RA^+^ CD27^+^) and memory (CD45RA^−^ CD27^+^) CD4 (CD3^+^ CD4^+^) or CD8 (CD3^+^ CD4^−^) T cells. (**b**) The Median Fluorescence Intensity (MFI) of t-STAT1 in the different T cell subsets was compared before and after treatment and viremia suppressed to <50 copies/ml using a Wilcoxon signed-rank test.

To test this hypothesis, we first studied a longitudinal cohort of patients to determine the levels of t-STAT1 before and after combination anti-retroviral therapy (cART) and suppressed viremia to <50 copies/ml. Following therapy, CD4 T cell counts increased from 198 to 264 cells/µl (Table 1). The analysis of the intracellular expression of t-STAT1 in naïve (CD45RA^+^ CD27^+^) and memory (CD45RA^−^ CD27^+^) CD4 and CD8 T cell subsets by flow cytometry showed that both naïve and memory CD4 T cells expressed high levels of t-STAT1 prior to therapy that were significantly reduced with treatment (*p*<0.01) (Fig. 1). Similar results were observed in naïve CD8 T cells (*p*<0.01). Memory CD8 T cells expressed lower levels of t-STAT1 than naïve CD8 T cells and their expression was also further reduced by treatment (*p* = 0.02) (Fig. 1). These results indicate that levels of t-STAT1 are upregulated during HIV infection. Additionally, the differential expression of t-STAT1 in CD4 and CD8 memory T cells suggests potential differences in their responses to Type-I IFNs.

**Table 1:** Characteristics of longitudinal study participants.

Patients (n = 10)	Pre-cART	>6 months on cART
**HIV RNA (copies/ml)**	(1)76081 (49209–160298)	(2)<50
**Months viral load <50 copies/ml**	-	11 (9–20)
**CD4 count (cells/µl)**	198 (121–240)	264 (243–295)
**CD8 count (cells/µl)**	992 (611–1680)	601 (470–1053)

Values are presented as median (IQR).

(1) Figure 1 (Patients VL >30 k copies/ml).

(2) Figure 1 (Patients VL <50 copies/ml).

### IL-7 enhances IFN-α responsiveness by modulating expression levels of t-STATs in T cells

**Figure 2:**
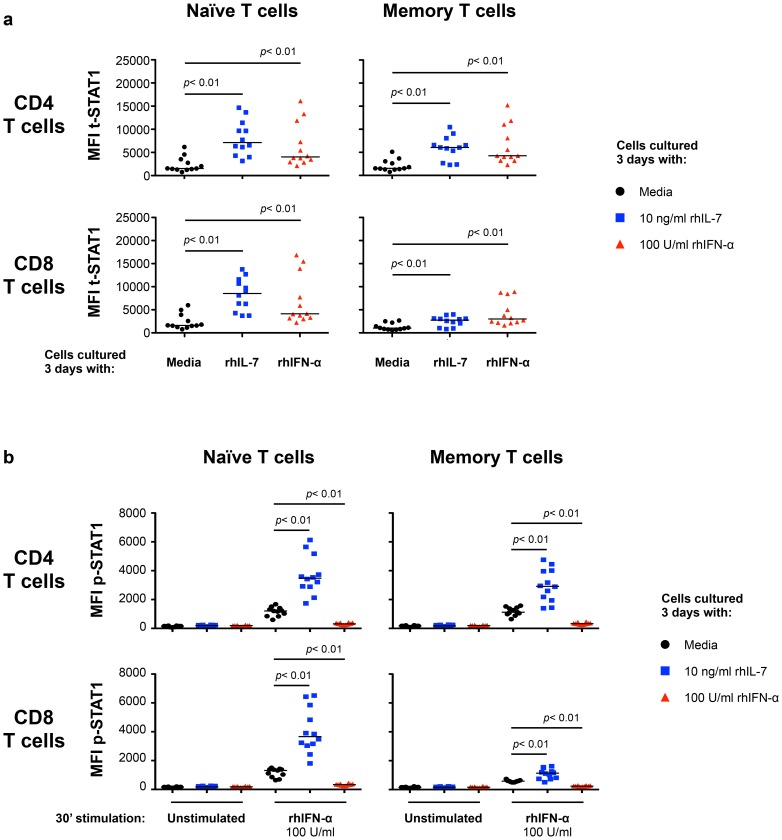
*In vitro* culture with IL-7 increases IFN-α-induced STAT1 activation (p-STAT1). PBMCs from healthy donors (n = 12) were cultured 3 days in media alone (black symbols), rhIL-7 (10 ng/ml; blue symbols) or rhIFN-α (100 U/ml; red symbols). After 3 days of culture, the cells were harvested, washed and rested overnight. Rested cells were stimulated *in vitro* with rhIFN-α (100 U/ml) for 30 minutes and analyzed for intracellular expression of t-STAT1 (**a**) and phosphorylated STAT1 (**b**) in naïve and memory T cell subsets. A Wilcoxon signed-rank test was performed for comparisons of the MFI of t-STAT1 and p-STAT1 between groups.

To define the mechanisms involved in modulating t-STAT1 levels during HIV infection, we examined the role of two cytokines associated with CD4 T cell lymphopenia (IL-7) [16], [17] and viral replication (IFN-α) [10], [14], [15]. PBMCs from healthy donors cultured with IL-7 for 3 days showed significant increases in t-STAT1 levels in naïve and memory CD4 T cells and naïve CD8 T cells when compared to cells cultured with media alone (*p*<0.01) (Fig. 2a and Fig. S1a, b). Consistent with the observations from the longitudinal cohort of patients (Fig. 1), the smallest increases were seen in memory CD8 T cells (*p*<0.01) (Fig. 2a).

We next tested the ability of cells cultured with IL-7 or IFN-α to respond to further *in vitro* stimulation with IFN-α. T cells cultured with IL-7 showed enhanced phosphorylation of STAT1 (p-STAT1) in response to *in vitro* IFN-α stimulation (Fig. 2b and Fig. S1c). In contrast, cells cultured for 3 days with IFN-α were refractory to further *in vitro* stimulation with IFN-α and did not show phosphorylation of STAT1 (Fig. 2b and Fig. S1c).

**Figure 3:**
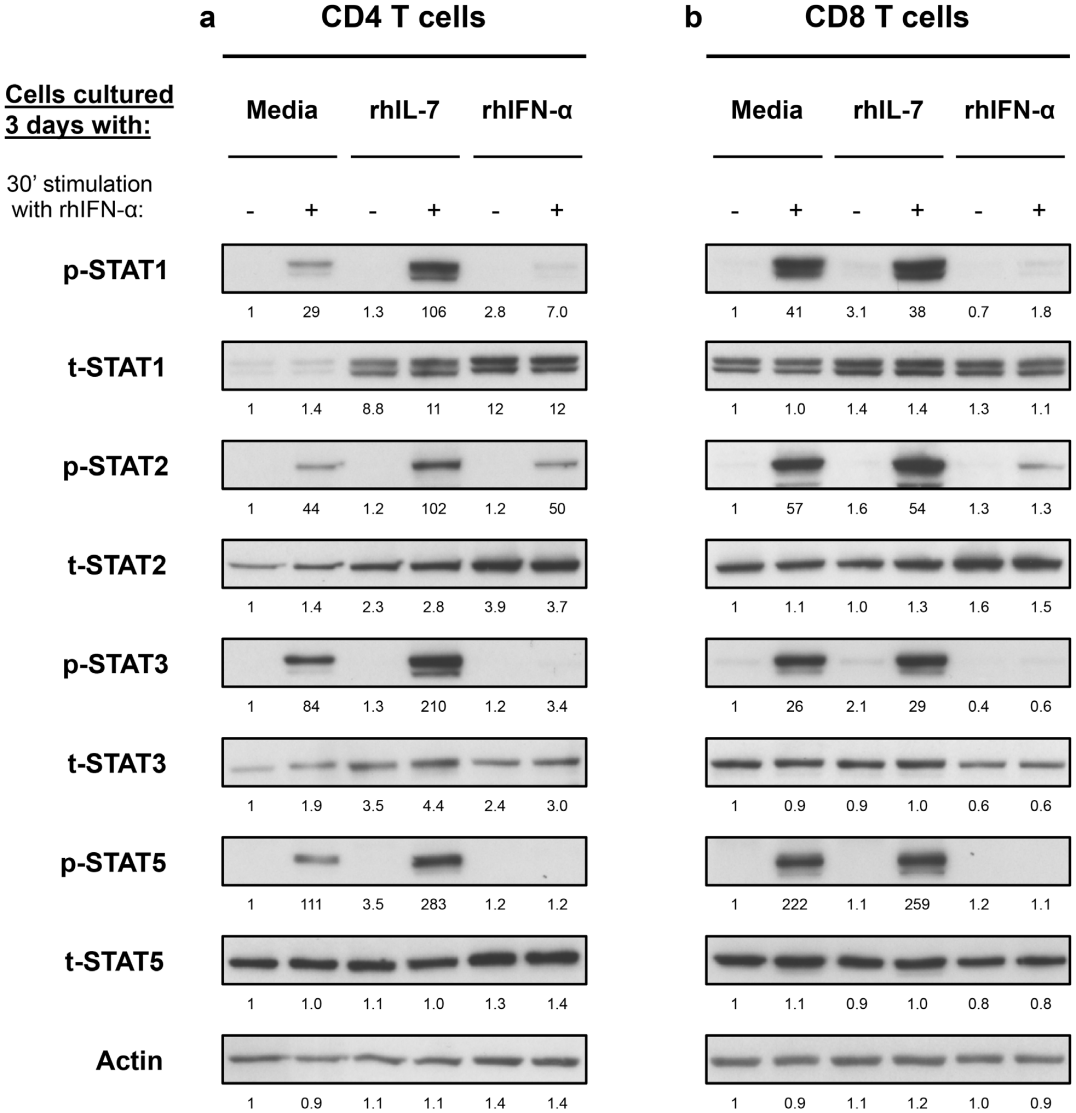
*In vitro* culture with IL-7 increases IFN-α-induced activation of STAT1, STAT2 and STAT3 in CD4 but not CD8 T cells. Isolated CD4 (**a**) and CD8 (**b**) T cells from a healthy donor were cultured and treated as described in Figure 2. Cell lysates were analyzed by Western blotting with antibodies specific to p-STAT1, t-STAT1, p-STAT2, t-STAT2, p-STAT3, t-STAT3, p-STAT5 and t-STAT5. An antibody to actin was used to confirm even protein loading. Numbers represent the ratio of the densitometry values of band densities on western blots calculated using the values of the unstimulated cells cultured for 3 days with media only as the baselines. Results are representative of at least 3 different donors.

These results suggested interplay between IL-7 and Type-I IFN in T cells that is mediated by the levels of STAT1 induction and subsequent phosphorylation. We next examined the effect of these cytokines on other STATs known to be involved in Type-I IFN signaling pathway (STAT2, -3 and -5) [13]. Freshly isolated CD4 T cells from healthy volunteers cultured for 3 days with IL-7 showed 8.3-, 2.1- and 2.7-fold increases in t-STAT1, t-STAT2 and t-STAT3 respectively (Fig. 3a). No changes were noted in total levels of STAT5. Cells cultured with IL-7 and then stimulated with IFN-α exhibited 3.7-, 2-, 2.5- and 2.5-fold increases in p-STAT1, -2, -3 and -5 respectively. No such effects of IL-7 were observed on total CD8 T cells likely due to the predominance of CD8 memory cells (Fig. 3b). These changes in CD4 T cells were mainly attributed to the modulation of t-STAT expression levels since IL-7 had no significant effect on the expression of IFNAR1 on T cells (Fig. S2). In contrast, cells initially cultured in the presence of Type-I IFN were generally refractory to further *in vitro* stimulation with IFN-α and phosphorylated only small amounts of STAT proteins (Fig. 3).

These results demonstrate that IL-7 can induce upregulation of multiple components of the Type-I IFN signaling pathway leading to an enhanced response to IFN-α. Thus, in the context of HIV infection, the increased availability of IL-7 associated with HIV-induced CD4 T cell lymphopenia, could render CD4 T cells more susceptible to the chronic effects of Type-I IFNs.

### Higher levels of t-STAT1 in CD4 T cells are associated with lower CD4 T cell counts and higher IL-7 serum levels in HIV infected patients

**Figure 4:**
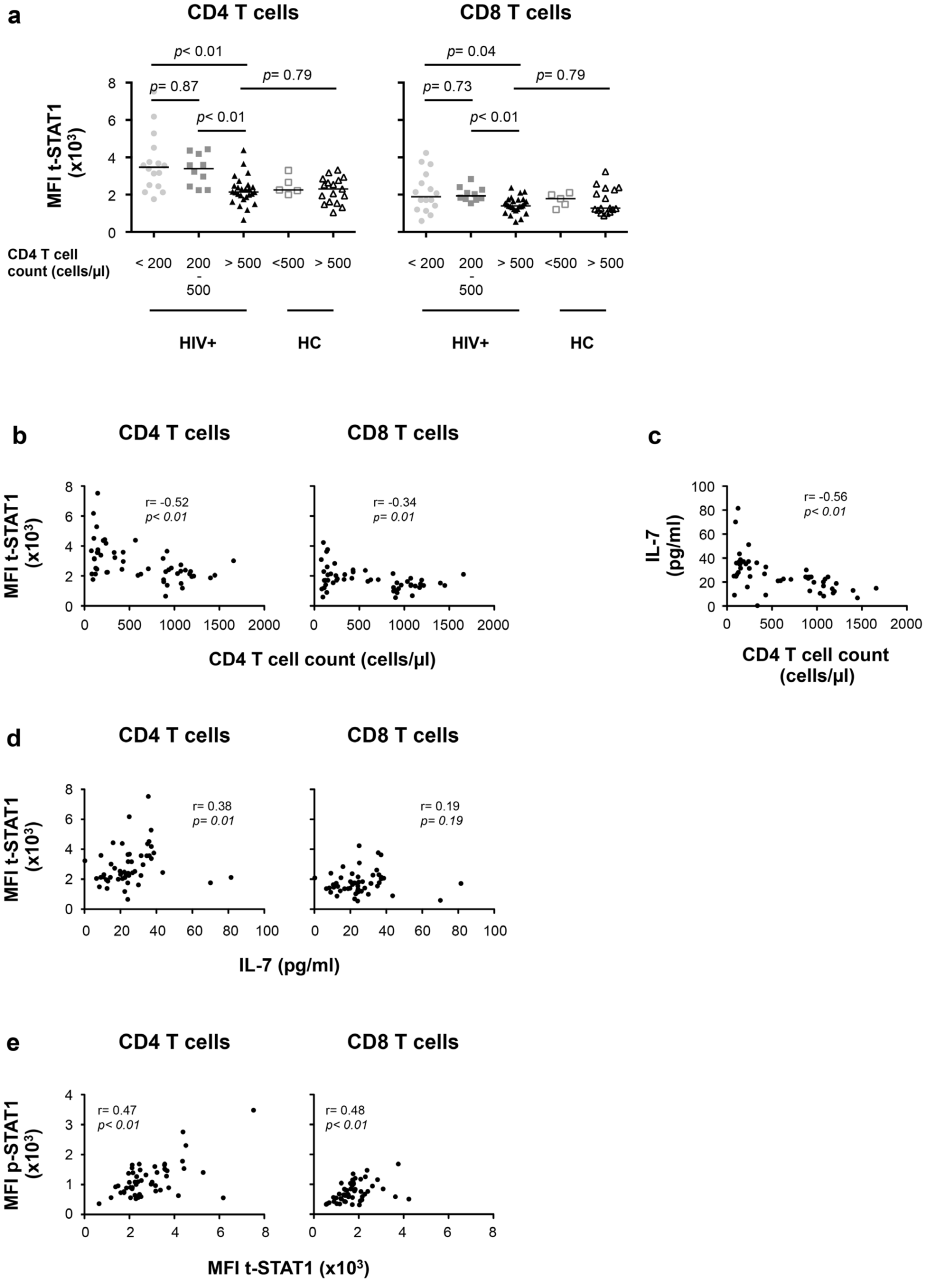
t-STAT1 expression is inversely associated with CD4 T cell counts and IL-7 serum levels in HIV-infected patients undergoing cART. PBMCs from healthy controls (HC, n = 22) and HIV-infected patients with viremia suppressed to <50 copies/ml for median 17 months on cART (HIV+, n = 53) were analyzed for t-STAT1 and p-STAT1 levels in total CD4 and CD8 T cell populations. Sera from the same patients were tested by ELISA for IL-7 levels. (**a**) The MFI of t-STAT1 in CD4 and CD8 T cells was compared between HIV+ and HC divided according to their CD4 T cell counts using a nonparametric Mann-Whitney test. (**b**) Relationship between t-STAT1 levels and CD4 T cell counts. (**c**) Relationship between IL-7 serum levels and CD4 T cell counts. (**d**) Relationship between t-STAT1 expression and IL-7 serum levels. (**e**) Relationship between STAT1 phosphorylation after *in vitro* stimulation with IFN-α and t-STAT1 levels. The correlations between the levels of t-STAT1, p-STAT1, IL-7 and CD4 T cell counts were analyzed with the non-parametric Spearman test.

We next evaluated the contributions of *in vivo* lymphopenia (CD4 T cell counts) and serum levels of IL-7 to t-STAT1 expression and activation (p-STAT1) in patients with chronic HIV infection. To limit the contributions of viremia, we studied a cohort of patients receiving cART who had HIV-RNA levels of <50 copies/ml for more than 9 months (IQR 9–35 months, Table 2). Levels of t-STAT1 and detection of p-STAT1 after *in vitro* stimulation with IFN-α were analyzed by flow cytometry. CD4 T cells from patients with CD4 counts <200 and between 200–500 cells/µl showed significantly higher levels of t-STAT1 expression when compared with those patients with CD4 counts >500 cells/µl (*p*<0.01). The latter showed t-STAT1 expression levels similar to those observed in healthy controls (Fig. 4a). Although significant, less marked differences were observed in CD8 T cells as a function of CD4 counts (Fig. 4a). In addition, an inverse correlation was noted between t-STAT1 levels in both CD4 and CD8 T cells and CD4 T cell counts (r = −0.52, *p*<0.01 and r = −0.34, *p*<0.01 respectively) (Fig. 4b). Serum levels of IL-7 were negatively associated with CD4 T cell counts (r = −0.56, *p*<0.01) (Fig. 4c) and positively associated with t-STAT1 levels in CD4 (r = 0.38, *p* = 0.01) but not CD8 T cells (Fig. 4d). Because of the association between sustained expansion of CD8 T cells and immune activation in patients with suppressed viremia [18], we next examined the relationship between t-STAT1 expression and IL-7 serum levels with CD4/CD8 T cell ratio. A similar negative association was observed between serum levels of IL-7 and t-STAT1 in CD4 and CD8 T cells with CD4/CD8 T cell ratio (r = −0.56, *p*<0.01, r = −0.36, p<0.01 and r = −0.56, p<0.01, respectively) (Fig. S3a, b).

**Table 2:** Characteristics of cross-sectional data participants.

		HIV+ Patients			Healthy Controls		
		CD4 count (cells/µl)				CD4 count (cells/µl)	
	All (n = 53)	<200 (n = 16)	200–500 (n = 11)	>500 (n = 26)	All (n = 22)	<500 (n = 5)	>500 (n = 17)
**HIV RNA (copies/ml)**	<50	<50	<50	<50	-	-	-
**HIV-RNA <50 copies/ml (months)**	17 (9–35)	12 (7–16)	17 (9–36)	22 (9–44)	-	-	-
**CD4 count (cells/µl)**	432 (148–1001)	124 (102–146)	256 (239–417)	1001 (881–1135)	689 (517–1006)	342 (267–408)	824 (605–1127)
**CD8 count (cells/µl)**	960 (647–1215)	649 (472–944)	990 (843–1398)	1023 (724–1389)	372 (197–575)	198 (147–245)	497 (279–628)
**Ratio CD4/CD8 counts**	0.51 (0.24–0.98)	0.19 (0.11–0.27)	0.25 (0.20–0.50)	0.98 (0.72–1.31)	1.67 (1.36–2.82)	1.73 (1.36–2.28)	1.59 (1.35–3.14)

Values are presented as median (IQR).

The expression levels of t-STAT1 in T cells from HIV infected patients were associated with p-STAT1 levels after *in vitro* stimulation with IFN-α in both CD4 and CD8 T cells (r = 0.47, *p*<0.01 and r = 0.48, *p*<0.01, respectively) (Fig. 4e). Patients with CD4 counts of >500 cells/µl and healthy controls showed similar activation of STAT1 after *in vitro* stimulation with IFN-α (Fig. S3c).

Taken together, these results indicate that, in the setting of HIV infection, CD4 T cell lymphopenia can contribute to upregulating t-STAT1 expression in CD4 T cells and this subsequently leads to increased levels of p-STAT1 following exposure to IFN-α.

### Lymphopenia and chronic exposure to Type-I IFN alter CD4 T cell homeostasis in a murine model

To experimentally examine the interaction between lymphopenia and Type-I IFN *in vivo* and its potential impact in CD4 and CD8 T cell homeostasis, we performed a series of experiments in which lymphocytes were adoptively transferred into lymphopenic mice that were receiving chronically-administered IFN-α.

**Figure 5:**
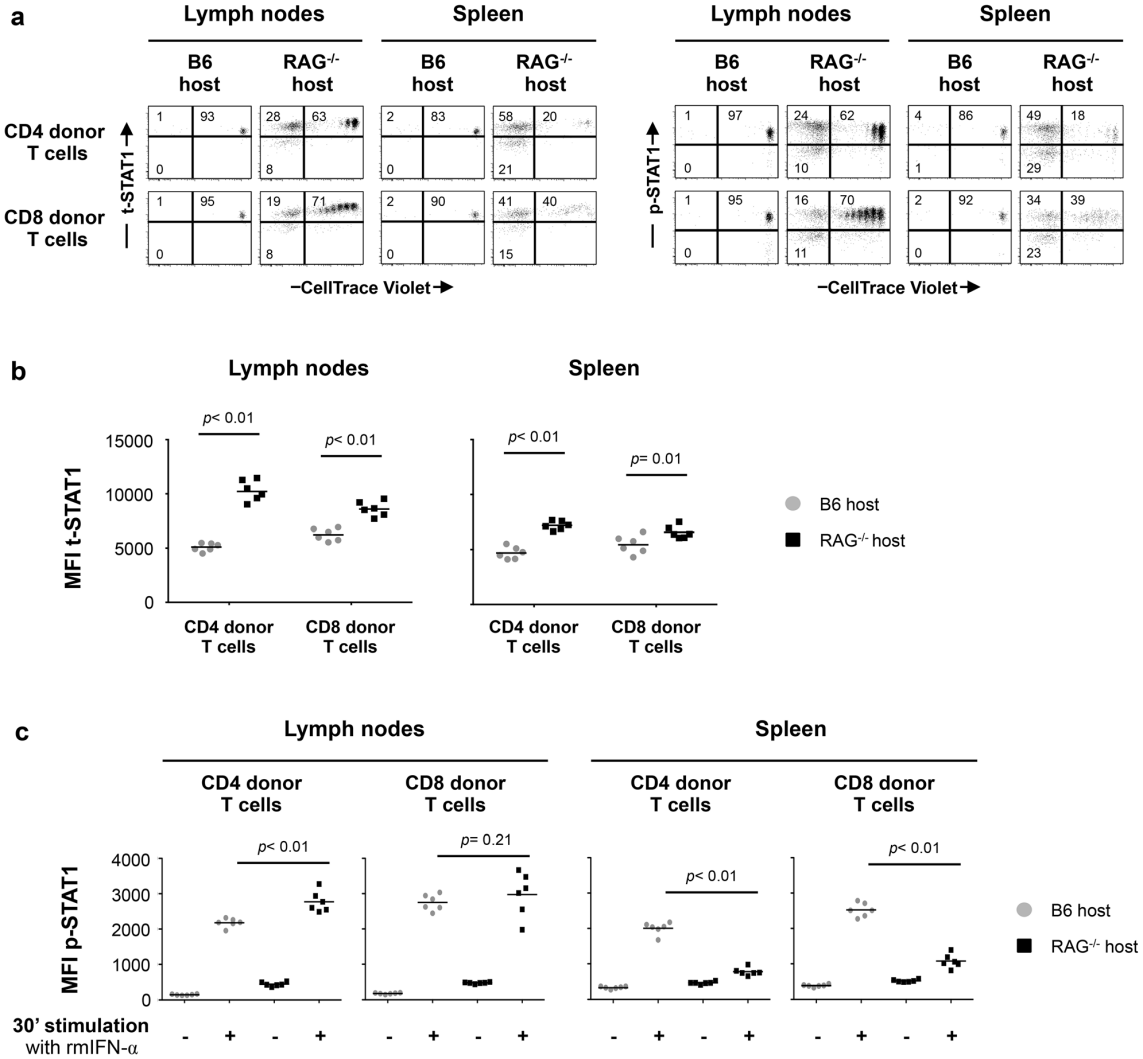
*In vivo* exposure to lymphopenic conditions enhances t-STAT1 expression and IFN-α responsiveness. Lymphoreplete B6 CD45.1 (n = 6) and lymphopenic RAG^−/−^ CD45.1 (n = 6) mice were injected intravenously (i.v.) with 9×10^6^ of CellTrace Violet-labeled lymph node (LN) cells isolated from congenic B6 CD45.2 mice. Analysis of transferred cells in LNs and spleen was performed on day 7 after transfer. The levels of t-STAT1 and p-STAT1 of donor T cells were evaluated by flow cytometry in LNs and spleen as function of CellTrace Violet Fluorescence after *in vitro* stimulation with rmIFN-α (500 U/ml). (**a**) The percentages of donor T cells CellTrace Violet**^+^** t-STAT1**^high^**, CellTrace Violet**^−^** t-STAT1**^high^**, CellTrace Violet**^−^** t-STAT1**^low^** and CellTrace Violet**^+^** p-STAT1**^high^**, CellTrace Violet**^−^** p-STAT1**^high^** and CellTrace Violet**^−^** p-STAT1**^low^** are indicated. The MFIs of t-STAT1 (**b**) and p-STAT1 (**c**) in CD4 and CD8 donor T cells seven days after adoptive transfer into replete B6 (gray symbols) and lymphopenic RAG^−/−^ (black symbols) hosts were compared using a nonparametric Mann-Whitney test. Data are from one representative experiment out of three, including an average of 5 mice per group.

Initially, we analyzed t-STAT1 and p-STAT1 expression in donor T cells transferred into lymphopenic or lymphoreplete mice. Lymph node (LN) cells from wild type C57BL/6 (B6 CD45.2 donor) mice were adoptively transferred into either congenic lymphoreplete B6.SJL (B6 CD45.1 host) or lymphopenic B6.SJL-[KO]RAG1 (RAG^−/−^ host) mice. At day 7 after transfer, both CD4 and CD8 donor T cells proliferated under lymphopenic conditions, whereas they did not divide when transferred into lymphoreplete hosts (Fig. 5a). Donor T cells transferred into lymphopenic RAG^−/−^ mice showed significantly increased levels of t-STAT1, in LNs as well as in the spleen, when compared to cells transferred into lymphoreplete B6 hosts (Fig. 5a, b). The lower upregulation of t-STAT1 in splenic cells was associated with an increased proportion of highly proliferating T cells with an effector memory and effector phenotype when compared to cells derived from the LNs (Fig. S4). In the LNs, increased levels of t-STAT1 were associated with enhanced phosphorylation of STAT1 in response to *in vitro* IFN-α stimulation in CD4 donor T cells (Fig. 5a, c). In contrast, lower levels of STAT1 phosphorylation were noted in splenic cells after *in vitro* stimulation with IFN-α when comparing cells that had been transferred into lymphopenic mice to those transferred into lymphoreplete hosts (Fig. 5c). Identical levels of t-STAT1 expression and activation after *in vitro* stimulation with IFN-α were observed in donor and recipient T cells from the lymphoreplete B6 host (Fig. S5). These data suggest that a lymphopenic environment can induce upregulation of t-STAT1 expression, leading to enhanced p-STAT1 following IFN-α stimulation.

**Figure 6:**
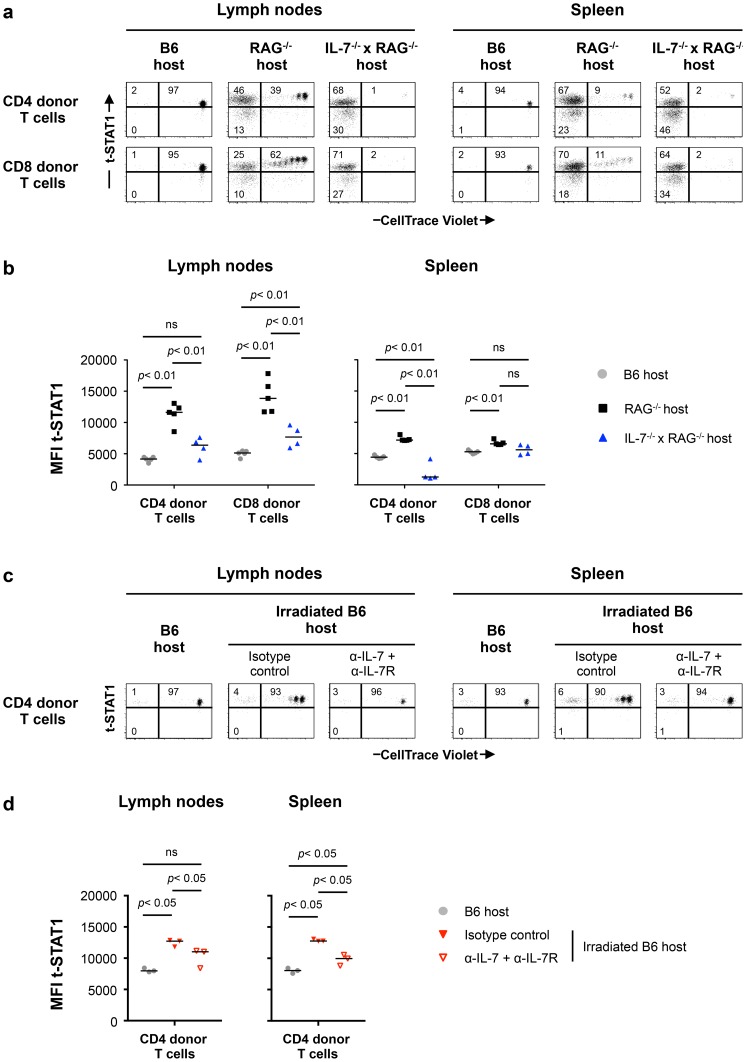
Lymphopenia-induced t-STAT1 upregulation in T cells is partially dependent on IL-7. (**a**) Lymphoreplete B6 CD45.1 (n = 5; gray symbols), lymphopenic RAG^−/−^ (n = 5; black symbols) and IL-7^−/−^ x RAG^−/−^ (n = 4; blue symbol) mice were injected i.v. with 9×10^6^ of CellTrace Violet-labeled LN cells isolated from congenic B6 CD45.2 mice. Analysis of transferred cells in LNs and spleen was performed on day 5 after transfer and levels of t-STAT1 of donor T cells were evaluated by flow cytometry as function of CellTrace Violet Fluorescence. The percentages of donor T cells CellTrace Violet**^+^** t-STAT1**^high^**, CellTrace Violet**^−^** t-STAT1**^high^** and CellTrace Violet**^−^** t-STAT1**^low^** are indicated. (**b**) The MFIs of t-STAT1 in CD4 and CD8 donor T cells in the different hosts were compared using a nonparametric Mann-Whitney test. Data are from one representative experiment out of three, including an average of 4 mice per group. (**c**) Non irradiated (n = 3, gray symbols) and irradiated B6 CD45.1 mice were adoptively transferred as described above. Irradiated B6 hosts were treated with either anti-IL-7 and anti-IL-7R (α-IL-7+α-IL-7R; n = 3, open red symbols) or the respective isotype matched control (n = 3, filled red symbols) mAbs. 5 days after transfer, mice were analyzed as described above and the percentages of donor T cells CellTrace Violet**^+^** t-STAT1**^high^**, CellTrace Violet**^−^** t-STAT1**^high^** and CellTrace Violet**^−^** t-STAT1**^low^** are indicated. (**d**) The MFI of t-STAT1 in donor T cells were compared between groups using a nonparametric Mann-Whitney test. Data are from one representative experiments out of three, including 3 mice per group.

Since IL-7 *in vitro* was able to upregulate t-STAT1, we next analyzed its contribution in the setting of lymphopenia. We performed adoptive cell transfer experiments into RAG^−/−^ and IL-7^−/−^ x RAG^−/−^ mice. Because IL-7 is critical for T cell survival, these experiments were analyzed 5 days after cell transfer. IL-7 deficiency in the RAG^−/−^ background reduced IL-7 dependent lymphopenia-induced slow proliferation and survival of the transferred cells (Fig. 6a, Fig. S6a) [19], [20]. In contrast, the rapid proliferation, which is known to be TCR dependent and IL-7 independent was not affected [21]. T cells derived from the LNs of IL-7 deficient RAG^−/−^ mice expressed reduced levels of t-STAT1 when compared with cells transferred into RAG^−/−^ hosts, although remained slightly higher than cells transferred into a lymphoreplete B6 host (Fig. 6a, b).

To eliminate the rapid TCR driven proliferation in the RAG^−/−^ background, we next assessed the role of IL-7 in sublethally irradiated hosts. In this model, the lymphopenia is not as severe as in RAG^−/−^ mice and transferred cells compete with the host cells during reconstitution by undergoing slow homeostatic proliferation which is IL-7 dependent [21], [22]. 24 hours after sublethal irradiation, mice were treated with either anti-IL-7 and anti-IL7R or isotype control mAbs before cell transfer [23], [24]. LNs and spleens were analyzed 5 days after transfer (Fig. 6c). In this model, donor cells undergoing homeostatic proliferation upregulated the levels of t-STAT1 when compared to cells transferred into a lymphoreplete B6 host (Fig. 6c, d). Treatment with anti-IL-7 and anti-IL7R mAbs blocked homeostatic proliferation. Because blockade of IL-7 signaling reduced survival of donor CD4 and especially CD8 T cells, in LNs and spleen, only CD4 donor T cells were analyzed (Fig. S6b) [19], [20]. Expression levels of t-STAT1 in donor T cells were reduced with α-IL-7+α-IL7R mAbs, however remained slightly higher than those observed in lymphoreplete B6 hosts (Fig. 6d). These results suggest that lymphopenia *in vivo* drives increased expression of t-STAT1 in CD4 T cells that is partially dependent on IL-7.

We next analyzed the impact of chronic exposure to IFN-α on T cell homeostasis. Lymphopenic RAG^−/−^ mice received LN cells from congenic B6 CD45.2 mice. Administration of recombinant murine (rm) IFN-α was started on day 5 after transfer. Mice were treated with therapeutic doses of 10,000 U of rmIFN-α, daily (∼4×10^5^ U/kg/day), for one month [25]–[27]. Control mice, which were otherwise identical to the experimental mice, were treated for the same period of time with an equivalent volume of PBS rather than rmIFN-α.

**Figure 7:**
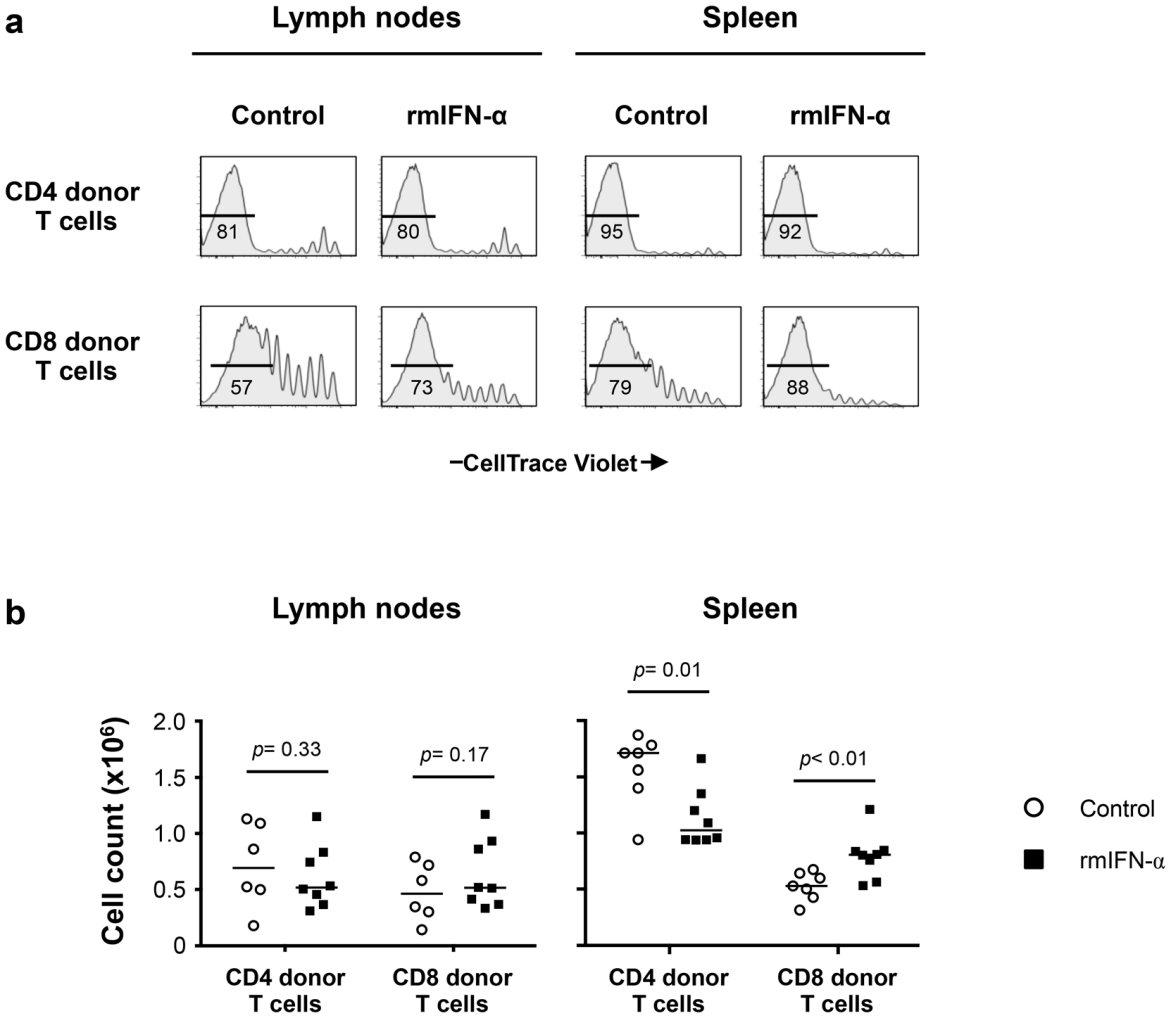
Continuous exposure to Type-I IFN under lymphopenic conditions leads to CD4 T cell depletion and CD8 T cell expansion. Lymphopenic RAG^−/−^ mice reconstituted with CellTrace Violet-labeled LN cells from congenic B6 CD45.2 mice were treated with either rmIFN-α (10,000 U per mouse, daily; n = 8) or PBS (n = 7). The numbers of CD4 and CD8 donor T cells in LNs and spleen were determined on day 35 after transfer. (**a**) Percentages of highly dividing CD45.2^+^ CD3^+^ CD4^+^ and CD8^+^ donor lymphocytes in LNs and spleen are shown. (**b**) Absolute numbers of CD4^+^ and CD8^+^ donor T cells in the lymphoid organs were enumerated following the treatment with rmIFN-α (black symbols) and PBS (open symbols). A nonparametric Mann-Whitney test was performed for comparisons of CD4 and CD8 T cell counts between groups. Data are from two representative experiments out of four, including an average of 4 mice per group.

Analysis of the CellTrace Violet profiles of CD4 and CD8 donor T cells showed extensive proliferation in LNs and spleens for both groups (Fig. 7a). We found no differences between the proliferation profiles of CD4 donor T cells from treated and non-treated animals. However, an increased proliferation of CD8 donor T cells was observed in both LNs and spleen of IFN-α treated mice, suggesting differences in the response to Type-I IFN by these two pools. Similar proportions of the naïve, central memory, effector memory and effector phenotype of CD4 donor T cells were observed in both LNs and spleen of the control and rmIFN-α treated animals (Fig. S7a, b). In contrast, in CD8 donor T cells, the proportion of naïve phenotype was reduced and increased proportions of central memory phenotype were observed in both organs of IFN-α treated mice (Fig. S7a, b).

While the absolute numbers of CD4 and CD8 donor T cells recovered from LNs did not differ substantially between IFN-α treated and control mice (Fig. 7b), significant decreases in CD4 donor T cell numbers were noted in the spleens of the rmIFN-α treated mice. In contrast, there was a significant increase in CD8 donor T cell numbers in the spleens of the treated mice (Fig. 7b). In addition, decreased CD4 T cell numbers in the spleens of IFN-α treated animals were primarily effector memory phenotype, suggesting that CD4 T cells may undergo activation induced cell death in this condition (Fig. S7c). Thus, chronic exposure to Type-I IFN in the setting of lymphopenia led to enhanced CD8 T cell expansion and impaired CD4 T cell homeostasis.

## Discussion

Patients with HIV infection show a unique form of immune activation leading to CD4 T cell depletion and CD8 T cell expansion. The CD4 T cell depletion observed in these patients is only partially explained by a direct cytopathic effect of the virus. The small number of actively infected cells at any given point suggests that other mechanisms may play a role [28], [29]. The mechanisms behind these extreme outcomes for both CD4 and CD8 T cells pools remain unresolved. Because CD4 T cells are under continuous homeostatic pressure, we hypothesized that these outcomes could be associated with differences in homeostatic regulation of these two pools and in their response to the combination of cytokines associated with lymphopenia and viral replication. In the present manuscript, we have shown that chronic exposure to Type-I IFN under lymphopenic conditions can lead to impaired CD4 T cell homeostasis resulting in diminished CD4 T cell counts in association with CD8 T cell expansion. This phenotype recapitulates the alterations of the CD4 and CD8 T cell pools seen in patients with HIV infection. Our data support a model in which CD4 T cells under lymphopenic conditions become more responsive to IFN-α by modulating the levels of STATs. This continuous stimulation of CD4 T cells may lead to activation induced cell death (AICD) and decreased survival. In contrast, the same set of stimuli lead to CD8 T cell expansion.

The different outcomes observed in the CD4 and CD8 T cell pools in the setting of untreated HIV infection are associated with distinct mechanisms that regulate the homeostasis of these pools. For instance, in healthy controls and HIV infected patients, CD4 T cell homeostatic proliferation is tightly associated with CD4 T cell counts. This association is weak in CD8 T cells [12]. The overall size of the CD4 T cell pool is highly controlled and does not undergo large expansions following antigenic exposure *in vivo*. In contrast, such expansions are common in the CD8 T cell pool.

Lymphopenia-induced proliferation is a mechanism triggered to maintain the CD4 T cell pool at a relatively constant size. IL-7 is a central homeostatic cytokine in this process that promotes T cell survival. Cells undergoing slow (IL-7 dependent) or fast (IL-7 independent) proliferation acquire new properties such as, memory phenotype and secretion of cytokines like IL-2 and IFN-γ [21], [30]. These effects are also observed in humans T cells *in vitro* [31] and in patients after bone marrow transplant [32]. Therefore, lymphopenia and increased availability of IL-7 generates a complex environment. In patients with HIV infection, the relatively weak association observed between IL-7 and levels of t-STAT1 suggests that lymphopenia in combination with other factors potentially driven by ongoing viral replication may play a role.

Several reports have shown that IL-7 upregulates expression of CD95 in T cells and IL-7 serum levels correlate with CD95 expression and increased susceptibility to CD95-induced apoptosis [33]–[35]. Our data extend these observations by demonstrating that IL-7 *in vitro* can also modulate the levels of STATs in T cells and thus modulate their responses to Type-I IFNs. This is an unexpected characteristic of IL-7 and suggests that this pathway may be advantageous in host defense against pathogens. Accordingly, recent reports in an animal model of acute influenza A virus suggested a role for IL-7 in adaptive immunity against viruses [36]. Similarly, administration of IL-7 in chronic LCMV infection model overcame infection and limited organ damage [37].

In contrast, in a scenario in which the host is chronically lymphopenic, such as HIV infection [16], [17], in association with an environment rich in Type-I IFNs maintained by viral replication, this interaction may lead to more immune activation and dysregulation of the homeostasis of the CD4 T cell pool. By modifying this relationship, such as by the administration of super physiological doses of IL-2 or IL-7 to patients with HIV infection receiving cART, one can see increased T cell counts [38]–[40]. Thus increasing the levels of homeostatic cytokine and reducing the inflammatory environment by cART may lead to improvement in T cell counts.

Type-I IFNs are important cytokines with anti-viral and regulatory functions [41]. They can inhibit thymopoiesis and B cell development [42] and can induce proliferation and exhaustion of hematopoietic stem cells [43]. The potential detrimental effects of chronic exposure to Type-I IFN in HIV/SIV pathogenesis have been largely reported. For example, Type-I IFN can induce CD4 T cell-TRAIL/DR5-mediated apoptosis [44]. Chronic exposure to Type-I IFN may have detrimental consequences on T cell homeostasis and survival [27], [44]–[48]. *In vivo*, HIV and SIV infection trigger a strong Type-I IFN response [10], [14], [15]. Paradoxically, the administration of super physiological doses of Type-I IFN have been shown to have both anti-tumor effects in the treatment of Kaposi sarcoma and anti-viral effects leading to reduction in HIV-RNA levels in patients with high CD4 T cell counts [49], [50]. These effects were associated with decreases in both CD4 and CD8 T cells.

Because of the potential detrimental effects of chronic Type-I IFN signaling in patients with HIV infection, blockade of this pathway has been explored as a potential therapeutic target. A recent report using an antagonist of TLR7/9 that blocks IFN-α production by plasmacytoid dendritic cells (pDCs) in SIV infected rhesus macaques had shown that pDCs are not the only source of IFN-α production in this infection model [51]. In addition, blockade of IFN-α production by pDCs did not prevent activation of T cells nor was it effective at reducing viral loads and had minimal effect on IFN-stimulated genes [51]. The anti-viral properties of IFN-α are best established and successfully used in the treatment of infectious diseases and cancer in humans [49], [50]. In this regard, it is not surprising that therapies blocking IFN-α signaling may compromise immunity against HIV and other pathogens.

In contrast to the anti-viral effects of Type-I IFNs, the immune modulatory properties of these cytokines are broad and less understood. For example, while IFN-β is used to treat the inflammatory autoimmune disease, multiple sclerosis, its mechanism of action in this setting is poorly understood [52]. The present model will help to dissect some of these properties of IFN-α signaling pathway in the setting of lymphopenia and may allow for identifying more direct targets of these immune modulatory functions.

IL-7 *in vitro* and lymphopenia *in vivo* differentially regulated expression of t-STATs in CD4 and CD8 T cells as well as their responses to Type-I IFN. Memory CD8 T cells from healthy controls and HIV-infected patients expressed lower levels of t-STAT1 than naïve CD8 T cells, suggesting differences in their responsiveness to Type-I IFN *in vivo* as a function of T cell phenotype and state of activation. Previous reports have suggested that differences in the expression levels and activation of STATs in human PBMCs may explain differences in the induction of apoptosis after exposure to Type-I IFNs [53]. In animal models of viral infection, it has been reported that T cell responsiveness to cytokines is controlled by their relative expression of the STAT transcription factors [54]. For instance, regulation of expression levels of t-STAT1 in CD8 T cells has been described as a mechanism by which CD8 T cells can escape the anti-proliferative effects of Type-I IFNs during LCMV infection [55] and basal and temporal changes in the expression levels of several STATs have been demonstrated as significant in shaping the immune responses in the LCMV model [56].

Altogether these findings suggest that during HIV infection, the interplay of lymphopenia and inflammation (Type-I IFN) may lead to alterations in the ways that CD4 and CD8 T cells respond to stimulation with Type-I IFNs. Further dissection of the relative roles of these forces during HIV infection may provide us with better approaches to correct the immunologic abnormalities seen in HIV infected patients.

## Materials and Methods

### Ethics statement

This human study was conducted according to the principles expressed in the Declaration of Helsinki. Patients were studied under a NIAID Institutional Review Board approved HIV clinical research study protocol in the NIAID/CCMD intramural program. Patients and healthy controls provided written informed consent for the collection of samples and subsequent analysis. Healthy volunteers were obtained through the NIH Blood Bank.

All animal work was conducted according to relevant national and international guidelines. The animal experiments were performed under a study protocol approved by the NIAID Animal Care and Use Committee.

### Patient and healthy volunteers

Patients and healthy controls were consented and studied in NIAID/CCMD intramural program IRB approved HIV clinical research studies. Healthy volunteers were obtained through the NIH Blood Bank. The majority of the patients studied had chronic HIV infection. The longitudinal cohort of HIV infected patients used to analyze the expression levels of t-STAT1 consisted of patients (n = 10) with viral loads of >30,000 copies/ml before starting cART and viremia suppressed to <50 copies/ml for more than 6 months (Table 1). The cohort of HIV infected patients used to assess the contributions of lymphopenia (CD4 counts and IL-7 serum levels) to t-STAT1 expression consisted of patients (n = 53) who had successfully suppressed viremia (viral load <50 copies/ml) with cART for more than 6 months. The patients' characteristics are described in Table 2 and viral infection history in Table S1.

### Cell culture and *in vitro* stimulation with IFN-α

PBMCs from healthy controls were obtained by Ficoll gradient centrifugation. CD4 and CD8 T cells were isolated by negative selection (Miltenyi Biotec) and cultured in X-VIVO 15 medium (Lonza) in absence or presence of the previously tested optimal concentration of recombinant human (rh) IL-7 (Peprotech) and rhIFN-α (PBL Biomedical laboratories). After 3 days of culture, the cells were harvested, washed with cytokine-free medium and adjusted at a concentration of 2×10^6^ cells/ml prior to resting overnight for subsequent *in vitro* stimulation with IFN-α (see below).

Cells that had been rested overnight were washed and labeled with live/dead (Invitrogen). Adjusted at a concentration of 2×10^6^ cells/ml, cells were then incubated for 30 minutes at 37°C and 5% CO_2_ with rhIFN-α (100 U/ml; PBL Biomedical laboratories) and p-STAT1 levels were analyzed by flow cytometry.

### Flow cytometry

IFN-α stimulations were stopped by fixation with 4% paraformaldehyde followed by a permeabilization step with 1∶1 methanol/acetone mix for 30 minutes on ice.

For human PBMCs, after three washes, cells were incubated for 10 minutes with 10 µg/ml human IgG (Sigma-Aldritch) to block potential Fc receptor binding and stained for 1 hour at room temperature with anti-CD3 Qdot 605 (Invitrogen, clone UCHT1), anti-CD4 Pacific blue (BD Biosciences, clone RPA-T4), anti-CD45RA PerCP-Cy5.5 (eBioscience, clone HI100), anti-CD27 PE (BD Biosciences, clone L128), anti-STAT1 N-terminus Alexa Fluor 647 (BD Biosciences, clone 1/Stat1), and anti-tyrosine 701-phosphorylated STAT1 Alexa Fluor 488 (Cell Signaling, clone 58D6).

For murine experiments (see below), donor T cells were detected by virtue of their CD45.2 expression. After three washes, mouse lymphocytes were stained for 30 minutes at room temperature with a mix of anti-STAT1 N-terminus Alexa Fluor 647 (BD Biosciences, clone 1/Stat1) and anti-tyrosine 701-phosphorylated STAT1 Alexa Fluor 488 (Cell Signaling, clone 58D6), followed by an additional 20 minutes incubation at room temperature with a mix of anti-CD3 V500 (BD Biosciences, clone 500A2), anti-CD4 PerCP-Cy5.5 (eBioscience, clone RM4-5), anti-CD8α eFluor 650NC (eBioscience, clone 53–6.7), anti-CD45.2 PE (eBioscience, clone 104) and anti-CD16/CD32 (BD Biosciences, clone 2.4G2). Full-minus-one controls were performed in the Alexa Fluor 647 and Alexa Fluor 488 channel for control of compensation spread [57].

The intensity of CellTrace Violet fluorescence was analyzed in fresh, unstimulated mouse lymphocytes incubated with anti-CD45.2 PE (eBioscience, clone 104), anti-CD3 APC (eBioscience, clone 17A2), anti-CD4 PerCP-Cy5.5 (eBioscience, clone RM4-5) and anti-CD8α eFluor 650NC (eBioscience, clone 53–6.7). This was performed for CD4 and CD8 donor T cells by gating respectively on CD45.2^+^ CD3^+^ CD4^+^ T cells and CD45.2^+^ CD3^+^ CD8^+^ T cells.

Human samples were collected on a BD LSR II and mouse samples were collected on a BD LSRFortessa using FACSDiva software. Data were subsequently analyzed using FlowJo (Tree Star).

### Western blot

CD4 and CD8 T cells from healthy controls were isolated by negative selection (Miltenyi Biotec), cultured for 3 days in absence or presence of rhIL-7 and rhIFN-α and further stimulated *in vitro* with rhIFN-α as described above. The stimulation was stopped by adding an equal volume of cold-temperature PBS containing phosphatase inhibitor to prevent de-phosphorylation of the activated STATs (Thermo Scientific). After three washes with cold-temperature PBS containing phosphatase inhibitor, the samples were stored at −20°C as dry pellets. Whole cell lysates were prepared from these samples with RIPA lysis buffer containing 50 mM Tris-HCl pH 7.5, 150 mM NaCl, 1% Nonidet P-40, 0.1% SDS, 0.5% Sodium Deoxycholate supplemented with protease inhibitor and phosphatase inhibitor cocktails (Pierce, Thermo Scientific) before use. Antibodies to STAT1 (clone E-23), STAT2 (clone C-20), STAT5 (clone C-17) were from Santa Cruz Biotechnology; and antibody to STAT3 was from Cell Signaling Technology. Phosphorylation-specific antibodies to STAT1 (Tyr701), STAT2 (Tyr690), STAT3 (Tyr705) and STAT5 (Tyr694) were from Cell Signaling Technology. After detection of the target protein, the membrane was stripped and reprobed with anti-beta-Actin antibody (clone mAbcam 8226; Abcam) to assess loading equivalency. Densitometric values of band densities on western blots were measured with ImageQuant TL 7.0 (GE Healthcare Life Sciences).

### Measurement of plasma IL-7 levels

Human IL-7 levels were quantified by ELISA, in cryopreserved plasma from HIV-infected individuals, using a commercial kit (R&D System) in accordance with the manufacturer's instructions. Samples were tested in duplicate.

### Mice, irradiation and adoptive T cell transfer

All experiments were approved by the NIAID Animal Care and Use Committee (ASP #: LIR5). B6.SJL-[KO]RAG1 (RAG^−/−^), B6.SJL (B6 CD45.1) and C57BL/6 (B6 CD45.2) mice were purchased from Taconic. IL-7^−/−^ x RAG^−/−^ mice were kindly provided by Dr. Scott K. Durum (NCI, Frederick, MD). All mice used in these studies were between 5 and 13 weeks of age.

B6 CD45.1 mice were sublethally irradiated (600 rads) utilizing a Cesium 137 source (J. L. Shepherd Mark 1). Under these conditions, depletion of host T cells was approximately 90% at 24 h after irradiation. Mice were used for adoptive transfer experiments 24 hours after irradiation.

For adoptive transfer studies, lymph node (LN) cells (2×10^7^ cells/ml) from B6 CD45.2 donor mice were labeled with 5 µM CellTrace Violet (Molecular Probes) in PBS for 10 minutes at 37°C. Lymphoreplete B6 CD45.1 and lymphopenic RAG^−/−^, IL-7^−/−^ x RAG^−/−^ and irradiated B6 CD45.1 recipient mice were injected i.v. with 9×10^6^ of B6 CD45.2 congenic cells.

### *In vivo* treatment with antibodies and rmIFN-α

B6 CD45.1 mice received every day alternately 0.5 mg/mouse of anti-IL-7 (clone M25; BioXCell) and 0.5 mg/mouse of anti-IL-7R (clone A7R34; BioXCell) by i.p. injection starting at 18 hours after irradiation and up to day 5. Control mice received the same amount of the matched isotype control mAbs (clone MPC-11, mouse IgG2b and clone 2A3, rat IgG2a, respectively; BioXCell).

For *in vivo* treatment with IFN-α, rmIFN-α (eBioscience: 10,000 U/mouse of rmIFN-α4 diluted in 0.2 ml PBS) was administered by subcutaneous (s.c.) injection every day starting on day 5 after adoptive transfer. Mice were treated for a month. Control mice were injected with 0.2 ml PBS.

Five to thirty-five days after transfer, spleen and a mixture of inguinal, axillary, cervical, mandibular, popliteal, mesenteric and pancreatic LN were excised. Spleen and LNs were processed separately to obtain single cell suspensions by mechanic disruption on Nitex filters in RPMI 1640 medium (Cellgro) containing 10% FCS, 55 µM β-mercaptoethanol, 2 mM L-glutamine and 50 µg/ml gentamicin (Complete media) at 4°C. After counting, cells from LNs and spleen were stained with the indicated Abs and/or stimulated *in vitro* with 500 U/ml of recombinant murine IFN-α4 (rmIFN-α; eBioscience) for 30 minutes at 37°C and 5% CO_2_, after being rested for at least 1 hour in a cytokine free complete medium.

### Statistical analysis method

For the human studies, Wilcoxon signed-rank tests were performed for within-group comparisons. Spearman's rank correlation coefficients were used to assess the association of t-STAT1 expression and plasma level of IL-7 with CD4 T cell counts, as well as the association among t-STAT1 expression, STAT1 phosphorylation after *in vitro* IFN-α stimulation, and IL-7 level in Figure 4. Nonparametric unpaired Mann-Whitney tests were used to analyze the data from the mouse experiments. Data were considered to be statistically different if the *p* value was ≤0.05.

## Supporting Information

Figure S1 ***In vitro*** **culture with IL-7 increases t-STAT1 expression and Type-I IFN responsiveness.** PBMCs from healthy donors were cultured 3 days in media alone, rhIL-7 (10 ng/ml) and rhIFN-α (100 U/ml). After 3 days of culture, the cells were harvested, washed and rested overnight. Rested cells were stimulated *in vitro* with rhIFN-α (100 U/ml) for 30 minutes and analyzed for intracellular expression of t-STAT1 and phosphorylated STAT1. (**a**) Gating strategy to assess t-STAT1 and p-STAT1 expression in T cell subsets, using CD27 and CD45RA as markers of naïve (CD45RA^+^ CD27^+^) and memory (CD45RA^−^ CD27^+^) CD4 (CD3^+^ CD4^+^) or CD8 (CD3^+^ CD4^−^) T cells. (**b**) Flow cytometric analysis of t-STAT1 expression (open histograms) or isotype control (shaded histograms). (**c**) Overlay histograms showing upregulation of staining for p-STAT1 after 30 minutes *in vitro* stimulation with rhIFN-α (red) compared with unstimulated cells (blue). Data from one representative donor out of twelve are presented.
(PDF)

Figure S2 **IFNAR1 expression on T cells is not affected by** ***in vitro*** **culture with IL-7.** PBMCs or isolated CD4 and CD8 T cells from a healthy donor were cultured as described in Figure S1. After 3 days of culture, the cells were harvested, washed and rested overnight. (**a**) Cell lysates obtained from isolated CD4 and CD8 T cells were analyzed by Western blotting with antibodies specific to IFNAR1 (Santa Cruz Biotechnology) and IL-7Rα (EMD Millipore). An antibody to actin (Abcam) was used to confirm even protein loading. Numbers represent the ratio of the densitometry values of band densities on western blots calculated using the values of the cells cultured 3 days with media only as baseline. Results are representative of 3 different donors. (**b**) PBMCs were analyzed by flow cytometry for surface expression of IFNAR1 (R&D) and IL-7Rα (BD, clone hIL7R-M21) in naïve and memory T cell subsets gated as described in Figure S1. Data from one representative donor out of five are presented. (**c**) The MFIs of IFNAR1 and IL-7Rα in the different T cell subsets were compared between culture conditions using a Wilcoxon signed-rank test.
(PDF)

Figure S3 **t-STAT1 expression and IL-7 serum levels are inversely associated with CD4/CD8 T cell ratio HIV-infected patients undergoing cART.** PBMCs from healthy controls (HC, n = 22) and HIV-infected patients (HIV+, n = 53) described in Figure 4 were analyzed for t-STAT1 and p-STAT1 levels in total CD4 and CD8 T cell populations. Sera from the same patients were tested by ELISA for IL-7 levels. (**a**) Relationship between t-STAT1 levels and CD4/CD8 T cell ratio. (**b**) Relationship between IL-7 serum levels and CD4/CD8 T cell ratio. The correlations between the levels of t-STAT1, IL-7 and CD4/CD8 T cell ratio were analyzed with the non-parametric Spearman test. (**c**) The MFI of p-STAT1 after *in vitro* stimulation with IFN-α in CD4 and CD8 T cells was compared between HIV+ and HC divided according to their CD4 T cell counts using a nonparametric Mann-Whitney test.
(PDF)

Figure S4 **Phenotype of proliferating donor T cells after transfer into lymphopenic RAG^−/−^ mice.** Seven days after transfer, Expression of CD44 (eBioscience, clone IM7), CD62L (eBioscience, clone MEL-14) and CD25 (eBioscience, clone PC61.5) on CD4^+^ and CD8^+^ donor T cells was assessed in LNs and spleen of mice from transferred RAG^−/−^ mice described in Figure 5. (**a**) The expression of CD44, CD62L and CD25 on gated CD45.2^+^ CD3^+^ CD4^+^ and CD8^+^ lymphocytes is presented as a function of CellTrace Violet fluorescence and percentages of donor T cells CellTrace Violet**^+^** CD44**^low^**, CellTrace Violet**^+^** CD44**^high^**, CellTrace Violet**^−^** CD44**^high^**, CellTrace Violet**^+^** CD62L**^high^**, CellTrace Violet**^−^** CD62L**^high^**, CellTrace Violet**^−^** CD44**^low^** and CellTrace Violet**^+^** CD25**^−^**, CellTrace Violet**^−^** CD25**^−^**, CellTrace Violet**^−^** CD25**^+^** are indicated. (**b**) The percentages of naïve (Tn: CD44^low^ CD62L^high^), central memory (Tcm: CD44^high^ CD62L^high^), effector memory (Tem: CD44^high^ CD62L^low^ CD25^−^) and effector (Teff: CD44^high^ CD62L^low^ CD25^+^) on donor CD4^+^ and CD8^+^ T cells in LN and spleen are indicated and presented as median. A Wilcoxon signed-rank test was performed for comparisons of the percentages of the different subsets between LNs and spleen. Data from one representative experiment out of three, including an average of 5 mice per group, are presented.
(PDF)

Figure S5 **Comparison of t-STAT1 and p-STAT1 expression between host and donor cells after adoptive transfer into lymphoreplete B6 mice.** (**a**) The levels of t-STAT1 and p-STAT1 of donor (gated CD45.2^+^ CD3^+^) and recipient (gated CD45.2^−^ CD3^+^) T cells from lymphoreplete B6 CD45.1 (n = 6) described in Figure 5 were evaluated in LNs and spleen after *in vitro* stimulation with rmIFN-α (500 U/ml). A nonparametric Mann-Whitney test was performed for the comparison of the MFI of t-STAT1 (**b**) and p-STAT1 (**c**) in CD4 and CD8 T cells between CD45.2^−^ CD3^+^ recipient (open gray symbols) and CD45.2^+^ CD3^+^ donor (solid gray symbols) T cells. Data from one representative experiment out of three, including an average of 5 mice per experiment, are presented.
(PDF)

Figure S6 **IL-7 blockade impairs donor T cell survival.** Absolute numbers of CD4^+^ and CD8^+^ donor T cells in the lymphoid organs of the transferred mice were enumerated at day five post transfer into (**a**) replete B6 CD45.1 (n = 5; gray symbols), lymphopenic RAG^−/−^ (n = 5; black symbols) and IL-7^−/−^ x RAG^−/−^ (n = 4; blue symbol) described in Figure 6a, b and (**b**) non irradiated (n = 3, gray symbols) and irradiated B6 hosts treated or not with anti-IL-7 and anti-IL-7R mAbs (α-IL-7+α-IL-7R; n = 3, open red symbols and Isotype control; n = 3, filled red symbols) described in Figure 6c, d. Data are from one representative experiments out of three, including 3 mice per group.
(PDF)

Figure S7 **Phenotype of donor T cells in lymphopenic mice chronically treated with IFN-α.** Thirty-five days after transfer, expression of CD44, CD62L and CD25 on CD4^+^ and CD8^+^ donor T cells was assessed in LNs and spleen of mice from the groups described in Figure 7. (**a**) Gating strategy to assess the proportion of naïve (Tn: CD44^low^ CD62L^high^), central memory (Tcm: CD44^high^ CD62L^high^), effector memory (Tem: CD44^high^ CD62L^low^ CD25^−^) and effector (Teff: CD44^high^ CD62L^low^ CD25^+^) on gated CD45.2^+^ CD3^+^ CD4^+^ and CD8^+^ lymphocytes in control and IFN-α treated animals (**b**) The percentages of naïve, central memory, effector memory and effector on gated CD45.2^+^ CD3^+^ CD4^+^ and CD8^+^ lymphocytes in control (open symbol) and IFN-α treated (black symbols) animals are indicated and presented as median. (**c**) Absolute numbers of donor T cell subsets in the lymphoid organs were enumerated following the treatment with rmIFN-α (black symbols) and PBS (open symbols). A nonparametric Mann-Whitney test was performed for comparisons between groups. Data from two representative experiments out of four, including an average of 4 mice per group, are presented.
(PDF)

Table S1 **Viral Infection history from HIV-infected patients described in** **Figure 1** **and** **Figure 4** **.**
(PDF)

## References

[1] Boyman O, Purton JF, Surh CD, Sprent J (2007) Cytokines and T-cell homeostasis. Curr Opin Immunol 19: 320–326. 17433869 10.1016/j.coi.2007.04.015

[2] Hakim FT, Cepeda R, Kaimei S, Mackall CL, McAtee N, et al (1997) Constraints on CD4 recovery postchemotherapy in adults: thymic insufficiency and apoptotic decline of expanded peripheral CD4 cells. Blood 90: 3789–3798. 9345067

[3] Mackall CL, Hakim FT, Gress RE (1997) Restoration of T-cell homeostasis after T-cell depletion. Semin Immunol 9: 339–346. 9405262 10.1006/smim.1997.0091

[4] Douek DC (2002) The contribution of the thymus to immune reconstitution after hematopoietic stem-cell transplantation. Cytotherapy 4: 425–426. 12473210 10.1080/146532402320776035

[5] Catalfamo M, Di Mascio M, Hu Z, Srinivasula S, Thaker V, et al (2008) HIV infection-associated immune activation occurs by two distinct pathways that differentially affect CD4 and CD8 T cells. Proc Natl Acad Sci U S A 105: 19851–19856. 19060209 10.1073/pnas.0810032105 PMC2596741

[6] Foulds KE, Zenewicz LA, Shedlock DJ, Jiang J, Troy AE, et al (2002) Cutting edge: CD4 and CD8 T cells are intrinsically different in their proliferative responses. J Immunol 168: 1528–1532. 11823476 10.4049/jimmunol.168.4.1528

[7] Catalfamo M, Le Saout C, Lane HC (2012) The role of cytokines in the pathogenesis and treatment of HIV infection. Cytokine & growth factor reviews 23: 207–214. 22738931 10.1016/j.cytogfr.2012.05.007 PMC3726258

[8] Douek D (2007) HIV disease progression: immune activation, microbes, and a leaky gut. Topics in HIV medicine : a publication of the International AIDS Society, USA 15: 114–117. 17720995

[9] Kovacs JA, Lempicki RA, Sidorov IA, Adelsberger JW, Herpin B, et al (2001) Identification of dynamically distinct subpopulations of T lymphocytes that are differentially affected by HIV. J Exp Med 194: 1731–1741. 11748275 10.1084/jem.194.12.1731 PMC2193579

[10] Rotger M, Dalmau J, Rauch A, McLaren P, Bosinger SE, et al (2011) Comparative transcriptomics of extreme phenotypes of human HIV-1 infection and SIV infection in sooty mangabey and rhesus macaque. J Clin Invest 121: 2391–2400. 21555857 10.1172/JCI45235 PMC3104754

[11] Hyrcza MD, Kovacs C, Loutfy M, Halpenny R, Heisler L, et al (2007) Distinct transcriptional profiles in ex vivo CD4+ and CD8+ T cells are established early in human immunodeficiency virus type 1 infection and are characterized by a chronic interferon response as well as extensive transcriptional changes in CD8+ T cells. J Virol 81: 3477–3486. 17251300 10.1128/JVI.01552-06 PMC1866039

[12] Catalfamo M, Wilhelm C, Tcheung L, Proschan M, Friesen T, et al (2011) CD4 and CD8 T cell immune activation during chronic HIV infection: roles of homeostasis, HIV, type I IFN, and IL-7. Journal of immunology 186: 2106–2116. 10.4049/jimmunol.1002000 PMC7394280 21257970

[13] Gadina M, Hilton D, Johnston JA, Morinobu A, Lighvani A, et al (2001) Signaling by type I and II cytokine receptors: ten years after. Curr Opin Immunol 13: 363–373. 11406370 10.1016/s0952-7915(00)00228-4

[14] Jacquelin B, Mayau V, Targat B, Liovat AS, Kunkel D, et al (2009) Nonpathogenic SIV infection of African green monkeys induces a strong but rapidly controlled type I IFN response. J Clin Invest 119: 3544–3555. 19959873 10.1172/JCI40093 PMC2786805

[15] Bosinger SE, Li Q, Gordon SN, Klatt NR, Duan L, et al (2009) Global genomic analysis reveals rapid control of a robust innate response in SIV-infected sooty mangabeys. J Clin Invest 119: 3556–3572. 19959874 10.1172/JCI40115 PMC2786806

[16] Napolitano LA, Grant RM, Deeks SG, Schmidt D, De Rosa SC, et al (2001) Increased production of IL-7 accompanies HIV-1-mediated T-cell depletion: implications for T-cell homeostasis. Nat Med 7: 73–79. 11135619 10.1038/83381

[17] Fry TJ, Connick E, Falloon J, Lederman MM, Liewehr DJ, et al (2001) A potential role for interleukin-7 in T-cell homeostasis. Blood 97: 2983–2990. 11342421 10.1182/blood.v97.10.2983

[18] Serrano-Villar S, Gutierrez C, Vallejo A, Hernandez-Novoa B, Diaz L, et al (2013) The CD4/CD8 ratio in HIV-infected subjects is independently associated with T-cell activation despite long-term viral suppression. J Infect 66: 57–66. 23046968 10.1016/j.jinf.2012.09.013

[19] Tan JT, Dudl E, LeRoy E, Murray R, Sprent J, et al (2001) IL-7 is critical for homeostatic proliferation and survival of naive T cells. Proc Natl Acad Sci U S A 98: 8732–8737. 11447288 10.1073/pnas.161126098 PMC37504

[20] Schluns KS, Kieper WC, Jameson SC, Lefrancois L (2000) Interleukin-7 mediates the homeostasis of naive and memory CD8 T cells in vivo. Nat Immunol 1: 426–432. 11062503 10.1038/80868

[21] Min B, Yamane H, Hu-Li J, Paul WE (2005) Spontaneous and homeostatic proliferation of CD4 T cells are regulated by different mechanisms. J Immunol 174: 6039–6044. 15879097 10.4049/jimmunol.174.10.6039

[22] Guimond M, Veenstra RG, Grindler DJ, Zhang H, Cui Y, et al (2009) Interleukin 7 signaling in dendritic cells regulates the homeostatic proliferation and niche size of CD4+ T cells. Nat Immunol 10: 149–157. 19136960 10.1038/ni.1695 PMC2713006

[23] Sudo T, Nishikawa S, Ohno N, Akiyama N, Tamakoshi M, et al (1993) Expression and function of the interleukin 7 receptor in murine lymphocytes. Proc Natl Acad Sci U S A 90: 9125–9129. 8415665 10.1073/pnas.90.19.9125 PMC47514

[24] Grabstein KH, Waldschmidt TJ, Finkelman FD, Hess BW, Alpert AR, et al (1993) Inhibition of murine B and T lymphopoiesis in vivo by an anti-interleukin 7 monoclonal antibody. J Exp Med 178: 257–264. 8315381 10.1084/jem.178.1.257 PMC2191094

[25] Gil MP, Ploquin MJ, Watford WT, Lee SH, Kim K, et al (2012) Regulating type 1 IFN effects in CD8 T cells during viral infections: changing STAT4 and STAT1 expression for function. Blood 120: 3718–3728. 22968462 10.1182/blood-2012-05-428672 PMC3488885

[26] Audige A, Hofer U, Dittmer U, van den Broek M, Speck RF (2011) Evaluation of the immunomodulatory and antiviral effects of the cytokine combination IFN-alpha and IL-7 in the lymphocytic choriomeningitis virus and Friend retrovirus mouse infection models. Viral Immunol 24: 375–385. 21929334 10.1089/vim.2011.0006

[27] Essers MA, Offner S, Blanco-Bose WE, Waibler Z, Kalinke U, et al (2009) IFNalpha activates dormant haematopoietic stem cells in vivo. Nature 458: 904–908. 19212321 10.1038/nature07815

[28] Douek DC (2003) Disrupting T-cell homeostasis: how HIV-1 infection causes disease. AIDS Rev 5: 172–177. 14598566

[29] Lane HC (2010) Pathogenesis of HIV infection: total CD4+ T-cell pool, immune activation, and inflammation. Top HIV Med 18: 2–6. 20305309

[30] Cho BK, Rao VP, Ge Q, Eisen HN, Chen J (2000) Homeostasis-stimulated proliferation drives naive T cells to differentiate directly into memory T cells. J Exp Med 192: 549–556. 10952724 10.1084/jem.192.4.549 PMC2193235

[31] Geginat J, Sallusto F, Lanzavecchia A (2001) Cytokine-driven proliferation and differentiation of human naive, central memory, and effector memory CD4(+) T cells. J Exp Med 194: 1711–1719. 11748273 10.1084/jem.194.12.1711 PMC2193568

[32] Fry TJ, Mackall CL (2005) The many faces of IL-7: from lymphopoiesis to peripheral T cell maintenance. J Immunol 174: 6571–6576. 15905493 10.4049/jimmunol.174.11.6571

[33] Fluur C, De Milito A, Fry TJ, Vivar N, Eidsmo L, et al (2007) Potential role for IL-7 in Fas-mediated T cell apoptosis during HIV infection. J Immunol 178: 5340–5350. 17404319 10.4049/jimmunol.178.8.5340

[34] Negredo E, Massanella M, Puig J, Perez-Alvarez N, Gallego-Escuredo JM, et al (2010) Nadir CD4 T cell count as predictor and high CD4 T cell intrinsic apoptosis as final mechanism of poor CD4 T cell recovery in virologically suppressed HIV-infected patients: clinical implications. Clinical infectious diseases : an official publication of the Infectious Diseases Society of America 50: 1300–1308. 20367229 10.1086/651689

[35] Sammicheli S, Dang Vu Phuong L, Ruffin N, Pham Hong T, Lantto R, et al (2011) IL-7 Promotes CD95-Induced Apoptosis in B Cells via the IFN-gamma/STAT1 Pathway. PloS one 6: e28629. 22194871 10.1371/journal.pone.0028629 PMC3237470

[36] Plumb AW, Patton DT, Seo JH, Loveday EK, Jean F, et al (2012) Interleukin-7, but not thymic stromal lymphopoietin, plays a key role in the T cell response to influenza A virus. PLoS One 7: e50199. 23189186 10.1371/journal.pone.0050199 PMC3506535

[37] Pellegrini M, Calzascia T, Toe JG, Preston SP, Lin AE, et al (2011) IL-7 engages multiple mechanisms to overcome chronic viral infection and limit organ pathology. Cell 144: 601–613. 21295337 10.1016/j.cell.2011.01.011

[38] Levy Y, Lacabaratz C, Weiss L, Viard JP, Goujard C, et al (2009) Enhanced T cell recovery in HIV-1-infected adults through IL-7 treatment. J Clin Invest 119: 997–1007. 19287090 10.1172/JCI38052 PMC2662568

[39] Sereti I, Dunham RM, Spritzler J, Aga E, Proschan MA, et al (2009) IL-7 administration drives T cell-cycle entry and expansion in HIV-1 infection. Blood 113: 6304–6314. 19380868 10.1182/blood-2008-10-186601 PMC2710926

[40] Kovacs JA, Lempicki RA, Sidorov IA, Adelsberger JW, Sereti I, et al (2005) Induction of prolonged survival of CD4+ T lymphocytes by intermittent IL-2 therapy in HIV-infected patients. J Clin Invest 115: 2139–2148. 16025158 10.1172/JCI23196 PMC1174914

[41] Garcia-Sastre A, Biron CA (2006) Type 1 interferons and the virus-host relationship: a lesson in detente. Science 312: 879–882. 16690858 10.1126/science.1125676

[42] Wang J, Lin Q, Langston H, Cooper MD (1995) Resident bone marrow macrophages produce type 1 interferons that can selectively inhibit interleukin-7-driven growth of B lineage cells. Immunity 3: 475–484. 7584138 10.1016/1074-7613(95)90176-0

[43] Sato T, Onai N, Yoshihara H, Arai F, Suda T, et al (2009) Interferon regulatory factor-2 protects quiescent hematopoietic stem cells from type I interferon-dependent exhaustion. Nat Med 15: 696–700. 19483695 10.1038/nm.1973

[44] Herbeuval JP, Hardy AW, Boasso A, Anderson SA, Dolan MJ, et al (2005) Regulation of TNF-related apoptosis-inducing ligand on primary CD4+ T cells by HIV-1: role of type I IFN-producing plasmacytoid dendritic cells. Proc Natl Acad Sci U S A 102: 13974–13979. 16174727 10.1073/pnas.0505251102 PMC1224361

[45] Boasso A, Shearer GM (2007) Chronic innate immune activation as a cause of HIV-1 immunopathogenesis. Clin Immunol 126: 235–242. 17916442 10.1016/j.clim.2007.08.015 PMC2275778

[46] Preble OT, Rook AH, Quinnan GV, Vilcek J, Friedman RM, et al (1984) Role of interferon in AIDS. Ann N Y Acad Sci 437: 65–75. 6100011 10.1111/j.1749-6632.1984.tb37123.x

[47] Heikenwalder M, Polymenidou M, Junt T, Sigurdson C, Wagner H, et al (2004) Lymphoid follicle destruction and immunosuppression after repeated CpG oligodeoxynucleotide administration. Nat Med 10: 187–192. 14745443 10.1038/nm987

[48] Hardy GA, Sieg SF, Rodriguez B, Jiang W, Asaad R, et al (2009) Desensitization to type I interferon in HIV-1 infection correlates with markers of immune activation and disease progression. Blood 113: 5497–5505. 19299650 10.1182/blood-2008-11-190231 PMC2689050

[49] Lane HC, Kovacs JA, Feinberg J, Herpin B, Davey V, et al (1988) Anti-retroviral effects of interferon-alpha in AIDS-associated Kaposi's sarcoma. Lancet 2: 1218–1222. 2903954 10.1016/s0140-6736(88)90811-2

[50] Tavel JA, Huang CY, Shen J, Metcalf JA, Dewar R, et al (2010) Interferon-alpha produces significant decreases in HIV load. Journal of interferon & cytokine research : the official journal of the International Society for Interferon and Cytokine Research 30: 461–464. 10.1089/jir.2009.0090 PMC2964361 20235638

[51] Kader M, Smith AP, Guiducci C, Wonderlich ER, Normolle D, et al (2013) Blocking TLR7- and TLR9-mediated IFN-alpha production by plasmacytoid dendritic cells does not diminish immune activation in early SIV infection. PLoS Pathog 9: e1003530. 23935491 10.1371/journal.ppat.1003530 PMC3723633

[52] Axtell RC, Raman C (2012) Janus-like effects of type I interferon in autoimmune diseases. Immunol Rev 248: 23–35. 22725952 10.1111/j.1600-065X.2012.01131.x PMC3383665

[53] van Boxel-Dezaire AH, Zula JA, Xu Y, Ransohoff RM, Jacobberger JW, et al (2010) Major differences in the responses of primary human leukocyte subsets to IFN-beta. J Immunol 185: 5888–5899. 20956346 10.4049/jimmunol.0902314 PMC3244975

[54] Levy DE, Darnell JE Jr (2002) Stats: transcriptional control and biological impact. Nature reviews Molecular cell biology 3: 651–662. 12209125 10.1038/nrm909

[55] Lecuroux C, Girault I, Urrutia A, Doisne JM, Deveau C, et al (2009) Identification of a particular HIV-specific CD8+ T-cell subset with a CD27+ CD45RO-/RA+ phenotype and memory characteristics after initiation of HAART during acute primary HIV infection. Blood 113: 3209–3217. 19098272 10.1182/blood-2008-07-167601

[56] Kallal LE, Biron CA (2013) Changing partners at the dance: Variations in STAT concentrations for shaping cytokine function and immune responses to viral infections. JAKSTAT 2: e23504. 24058795 10.4161/jkst.23504 PMC3670271

[57] De Rosa SC, Herzenberg LA, Roederer M (2001) 11-color, 13-parameter flow cytometry: identification of human naive T cells by phenotype, function, and T-cell receptor diversity. Nat Med 7: 245–248. 11175858 10.1038/84701

