# Supplementary figures and images for: Chronic Exposure to Type-I IFN under Lymphopenic Conditions Alters CD4 T Cell Homeostasis
Source: PLoS Pathog. 2014 Mar 6;10(3):e1003976. doi: 10.1371/journal.ppat.1003976 (PMC3946368; doi:10.1371/journal.ppat.1003976)

Figure S1

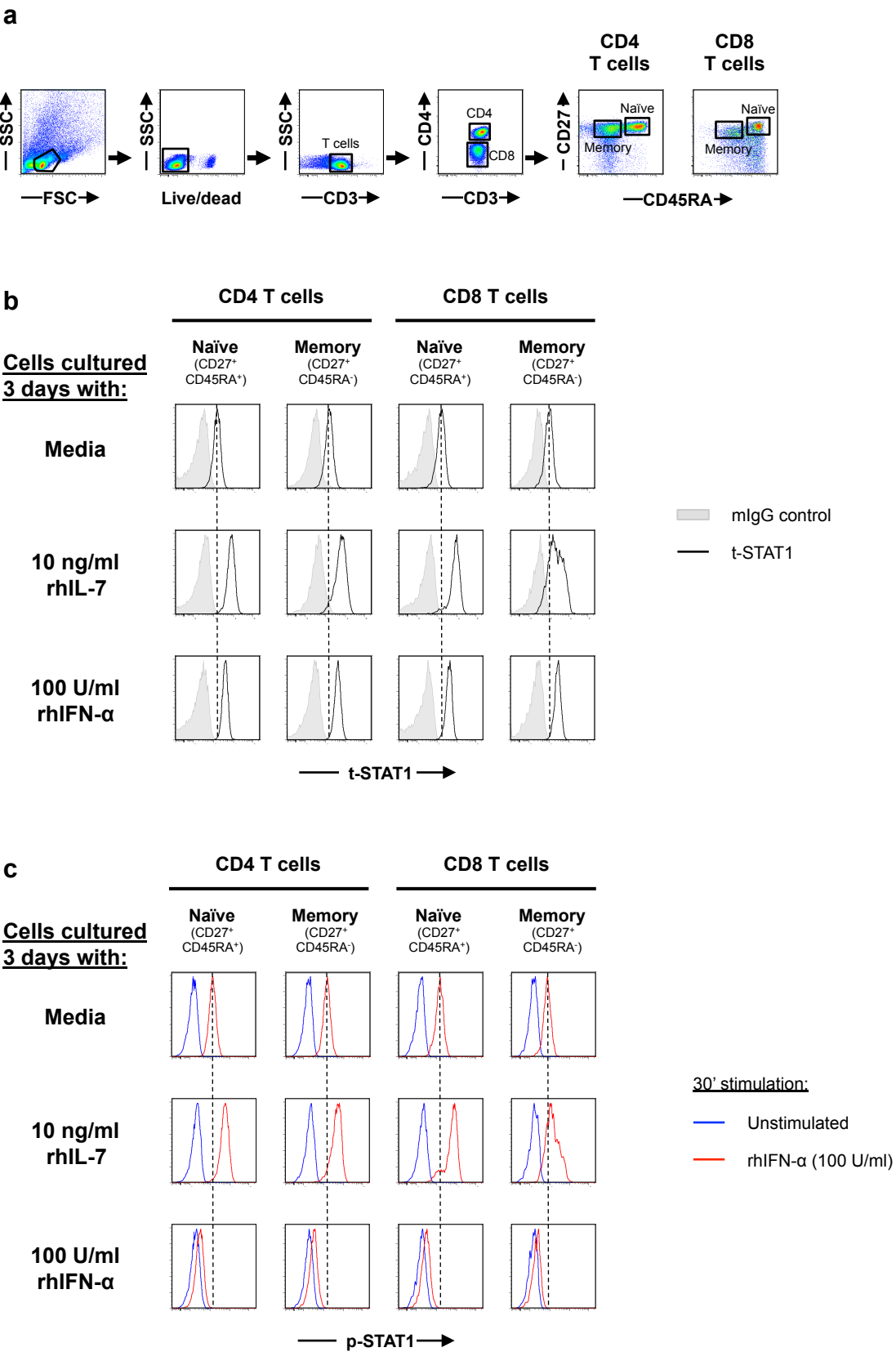

Supplement: Figure S1 — In vitro culture with IL-7 increases t-STAT1 expression and Type-I IFN responsiveness. PBMCs from healthy donors were cultured 3 days in media alone, rhIL-7 (10 ng/ml) and rhIFN-α (100 U/ml). After 3 days of culture, the cells were harvested, washed and rested overnight. Rested cells were stimulated in vitro with rhIFN-α (100 U/ml) for 30 minutes and analyzed for intracellular expression of t-STAT1 and phosphorylated STAT1. (a) Gating strategy to assess t-STAT1 and p-STAT1 expression in T cell subsets, using CD27 and CD45RA as markers of naïve (CD45RA+ CD27+) and memory (CD45RA− CD27+) CD4 (CD3+ CD4+) or CD8 (CD3+ CD4−) T cells. (b) Flow cytometric analysis of t-STAT1 expression (open histograms) or isotype control (shaded histograms). (c) Overlay histograms showing upregulation of staining for p-STAT1 after 30 minutes in vitro stimulation with rhIFN-α (red) compared with unstimulated cells (blue). Data from one representative donor out of twelve are presented. (PDF) [file ppat.1003976.s001.pdf]

Figure S2

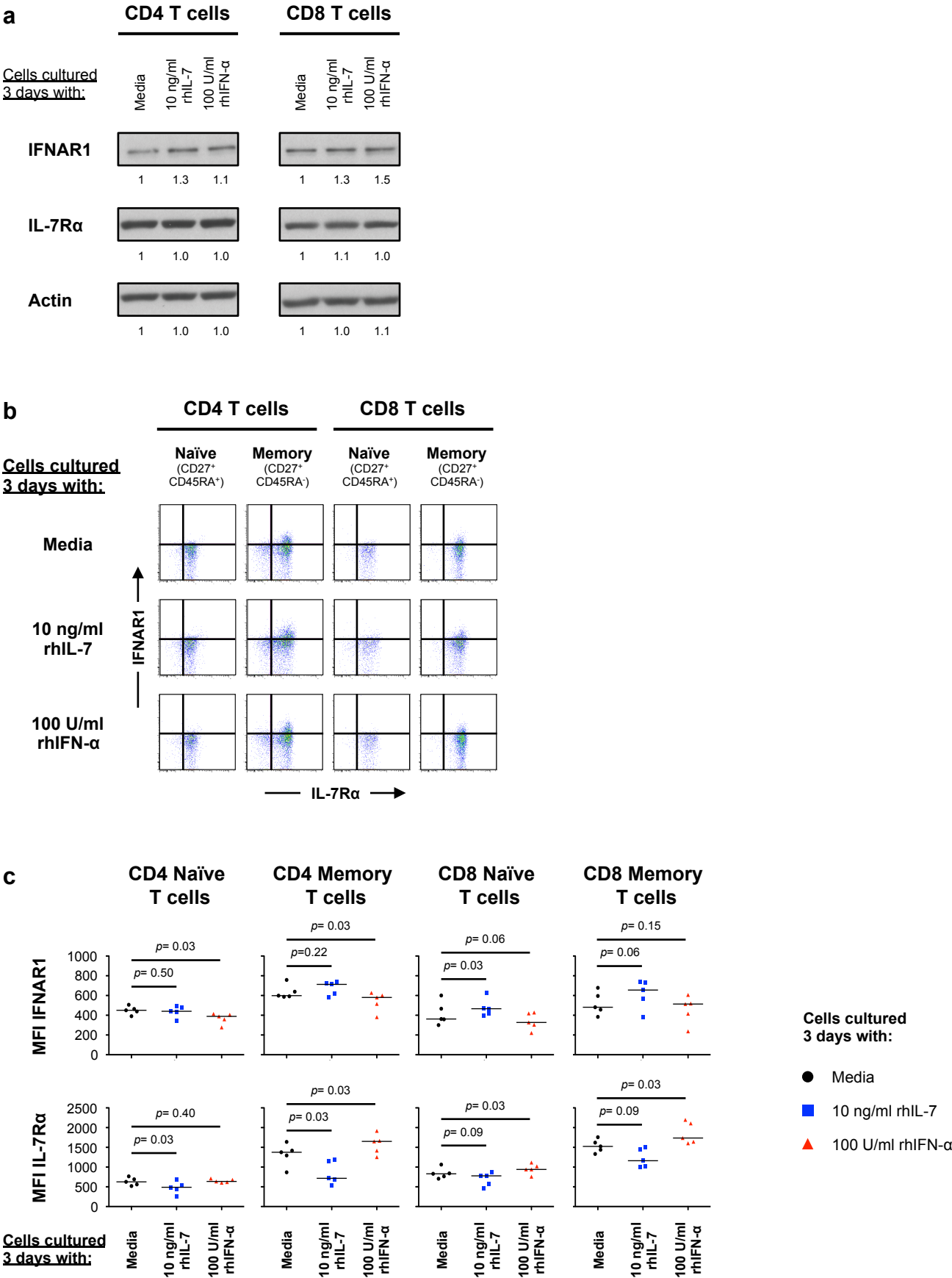

Supplement: Figure S2 — IFNAR1 expression on T cells is not affected by in vitro culture with IL-7. PBMCs or isolated CD4 and CD8 T cells from a healthy donor were cultured as described in Figure S1. After 3 days of culture, the cells were harvested, washed and rested overnight. (a) Cell lysates obtained from isolated CD4 and CD8 T cells were analyzed by Western blotting with antibodies specific to IFNAR1 (Santa Cruz Biotechnology) and IL-7Rα (EMD Millipore). An antibody to actin (Abcam) was used to confirm even protein loading. Numbers represent the ratio of the densitometry values of band densities on western blots calculated using the values of the cells cultured 3 days with media only as baseline. Results are representative of 3 different donors. (b) PBMCs were analyzed by flow cytometry for surface expression of IFNAR1 (R&D) and IL-7Rα (BD, clone hIL7R-M21) in naïve and memory T cell subsets gated as described in Figure S1. Data from one representative donor out of five are presented. (c) The MFIs of IFNAR1 and IL-7Rα in the different T cell subsets were compared between culture conditions using a Wilcoxon signed-rank test. (PDF) [file ppat.1003976.s002.pdf]

**Figure S3**

Le Saout et al.

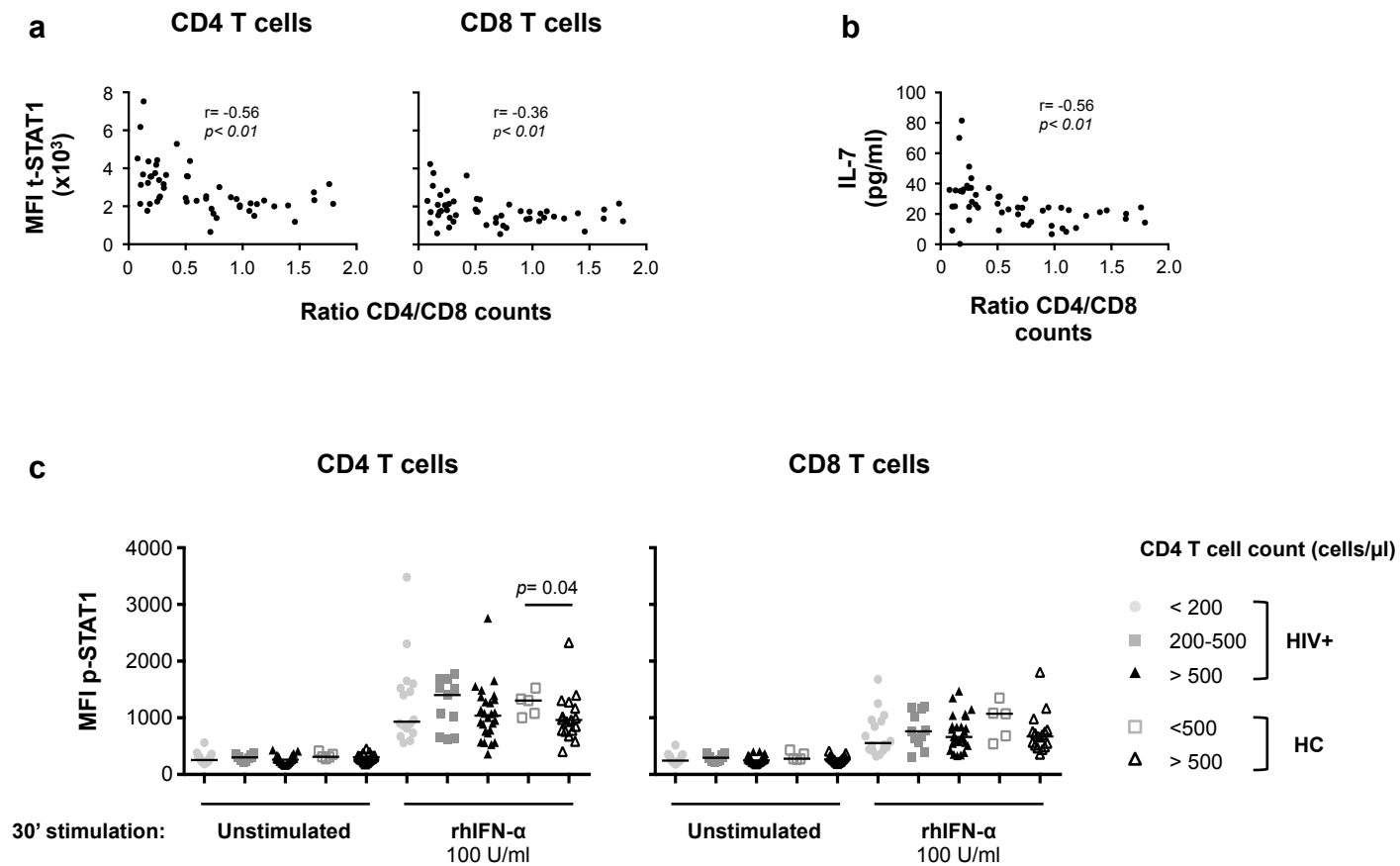

Supplement: Figure S3 — t-STAT1 expression and IL-7 serum levels are inversely associated with CD4/CD8 T cell ratio HIV-infected patients undergoing cART. PBMCs from healthy controls (HC, n = 22) and HIV-infected patients (HIV+, n = 53) described in Figure 4 were analyzed for t-STAT1 and p-STAT1 levels in total CD4 and CD8 T cell populations. Sera from the same patients were tested by ELISA for IL-7 levels. (a) Relationship between t-STAT1 levels and CD4/CD8 T cell ratio. (b) Relationship between IL-7 serum levels and CD4/CD8 T cell ratio. The correlations between the levels of t-STAT1, IL-7 and CD4/CD8 T cell ratio were analyzed with the non-parametric Spearman test. (c) The MFI of p-STAT1 after in vitro stimulation with IFN-α in CD4 and CD8 T cells was compared between HIV+ and HC divided according to their CD4 T cell counts using a nonparametric Mann-Whitney test. (PDF) [file ppat.1003976.s003.pdf]

**Figure S4**

Le Saout et al.

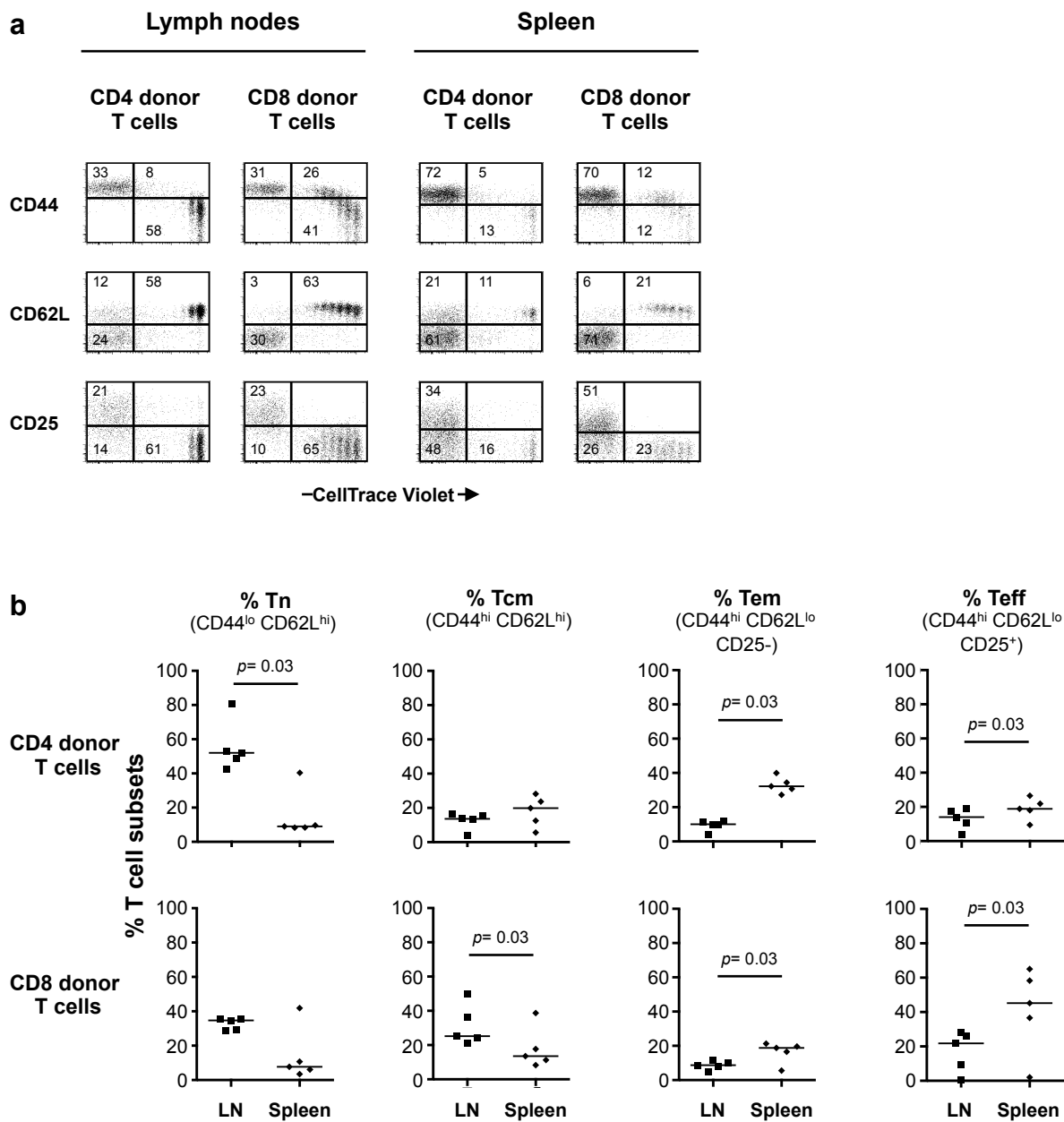

Supplement: Figure S4 — Phenotype of proliferating donor T cells after transfer into lymphopenic RAG−/− mice. Seven days after transfer, Expression of CD44 (eBioscience, clone IM7), CD62L (eBioscience, clone MEL-14) and CD25 (eBioscience, clone PC61.5) on CD4+ and CD8+ donor T cells was assessed in LNs and spleen of mice from transferred RAG−/− mice described in Figure 5. (a) The expression of CD44, CD62L and CD25 on gated CD45.2+ CD3+ CD4+ and CD8+ lymphocytes is presented as a function of CellTrace Violet fluorescence and percentages of donor T cells CellTrace Violet+ CD44low, CellTrace Violet+ CD44high, CellTrace Violet− CD44high, CellTrace Violet+ CD62Lhigh, CellTrace Violet− CD62Lhigh, CellTrace Violet− CD44low and CellTrace Violet+ CD25−, CellTrace Violet− CD25−, CellTrace Violet− CD25+ are indicated. (b) The percentages of naïve (Tn: CD44low CD62Lhigh), central memory (Tcm: CD44high CD62Lhigh), effector memory (Tem: CD44high CD62Llow CD25−) and effector (Teff: CD44high CD62Llow CD25+) on donor CD4+ and CD8+ T cells in LN and spleen are indicated and presented as median. A Wilcoxon signed-rank test was performed for comparisons of the percentages of the different subsets between LNs and spleen. Data from one representative experiment out of three, including an average of 5 mice per group, are presented. (PDF) [file ppat.1003976.s004.pdf]

**Figure S5**

Le Saout et al.

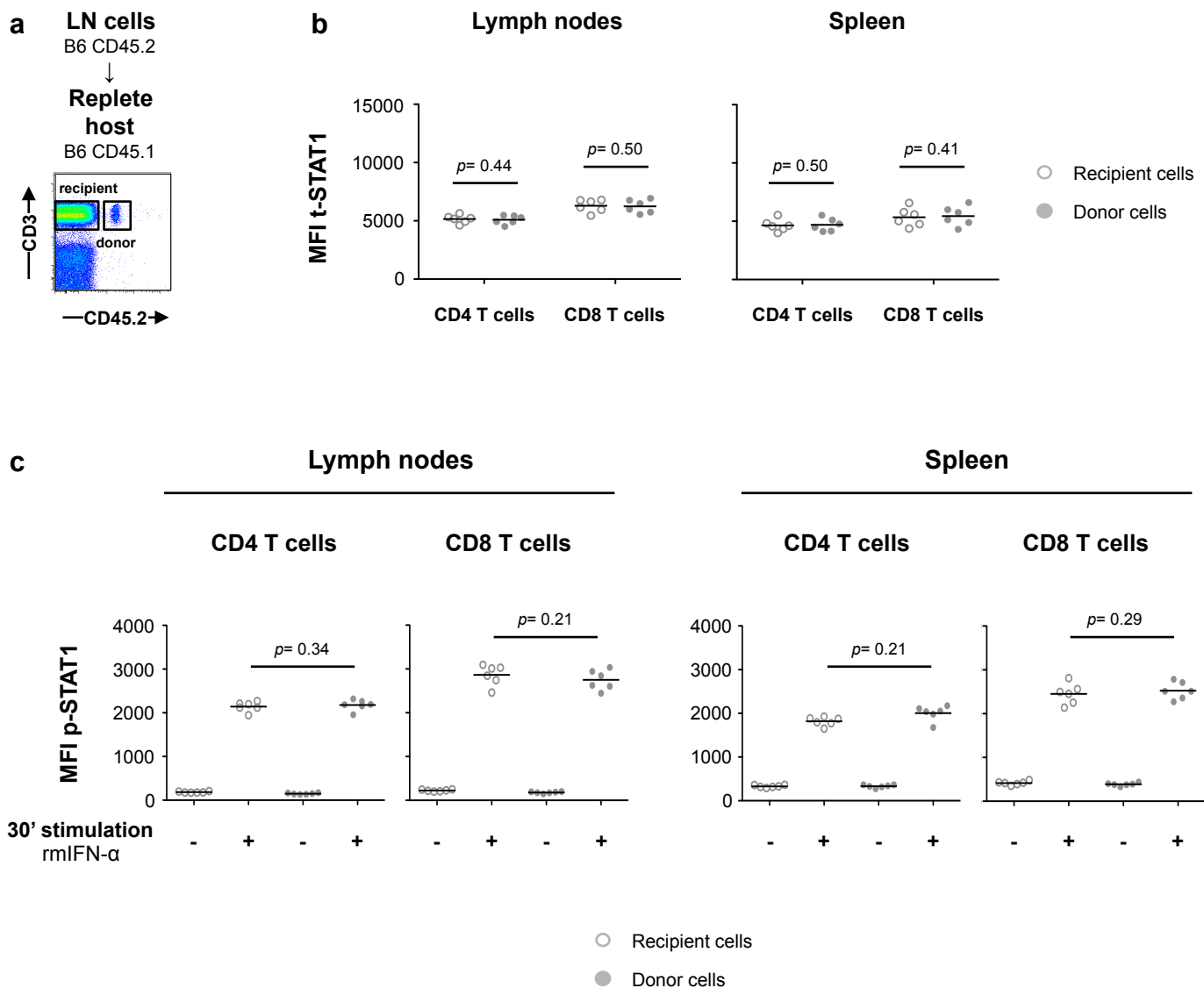

Supplement: Figure S5 — Comparison of t-STAT1 and p-STAT1 expression between host and donor cells after adoptive transfer into lymphoreplete B6 mice. (a) The levels of t-STAT1 and p-STAT1 of donor (gated CD45.2+ CD3+) and recipient (gated CD45.2− CD3+) T cells from lymphoreplete B6 CD45.1 (n = 6) described in Figure 5 were evaluated in LNs and spleen after in vitro stimulation with rmIFN-α (500 U/ml). A nonparametric Mann-Whitney test was performed for the comparison of the MFI of t-STAT1 (b) and p-STAT1 (c) in CD4 and CD8 T cells between CD45.2− CD3+ recipient (open gray symbols) and CD45.2+ CD3+ donor (solid gray symbols) T cells. Data from one representative experiment out of three, including an average of 5 mice per experiment, are presented. (PDF) [file ppat.1003976.s005.pdf]

Figure S6

Le Saout et al.

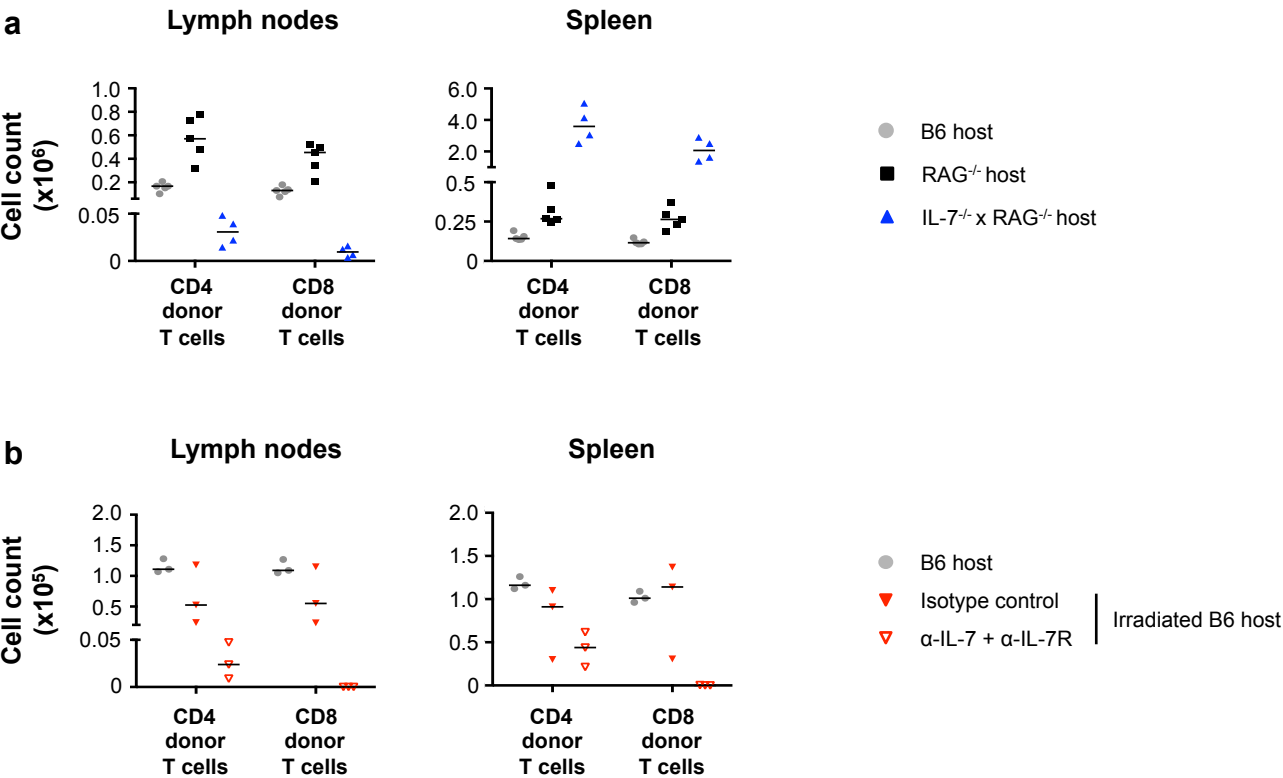

Supplement: Figure S6 — IL-7 blockade impairs donor T cell survival. Absolute numbers of CD4+ and CD8+ donor T cells in the lymphoid organs of the transferred mice were enumerated at day five post transfer into (a) replete B6 CD45.1 (n = 5; gray symbols), lymphopenic RAG−/− (n = 5; black symbols) and IL-7−/− x RAG−/− (n = 4; blue symbol) described in Figure 6a, b and (b) non irradiated (n = 3, gray symbols) and irradiated B6 hosts treated or not with anti-IL-7 and anti-IL-7R mAbs (α-IL-7+α-IL-7R; n = 3, open red symbols and Isotype control; n = 3, filled red symbols) described in Figure 6c, d. Data are from one representative experiments out of three, including 3 mice per group. (PDF) [file ppat.1003976.s006.pdf]

**Figure S7**

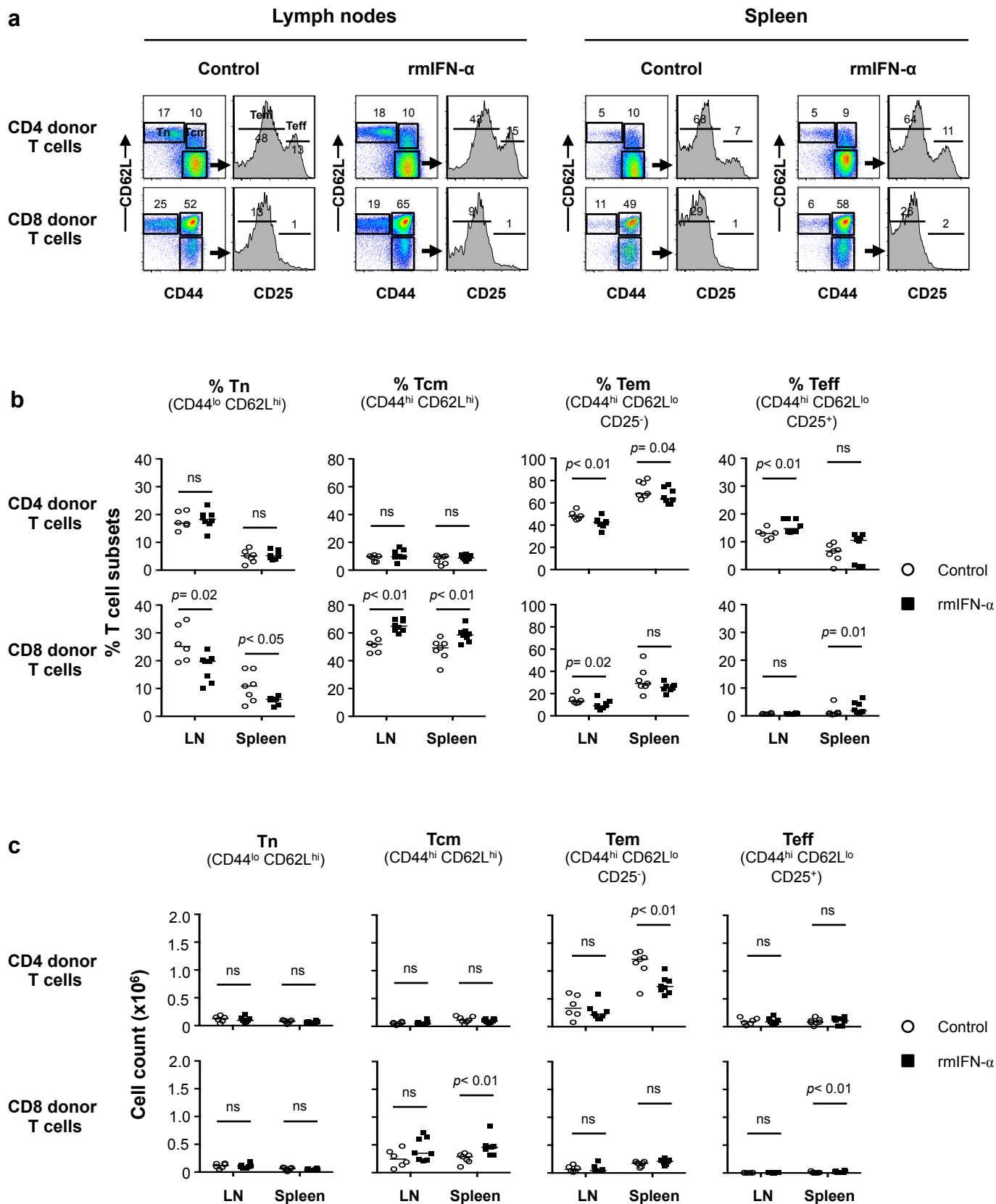

Supplement: Figure S7 — Phenotype of donor T cells in lymphopenic mice chronically treated with IFN-α. Thirty-five days after transfer, expression of CD44, CD62L and CD25 on CD4+ and CD8+ donor T cells was assessed in LNs and spleen of mice from the groups described in Figure 7. (a) Gating strategy to assess the proportion of naïve (Tn: CD44low CD62Lhigh), central memory (Tcm: CD44high CD62Lhigh), effector memory (Tem: CD44high CD62Llow CD25−) and effector (Teff: CD44high CD62Llow CD25+) on gated CD45.2+ CD3+ CD4+ and CD8+ lymphocytes in control and IFN-α treated animals (b) The percentages of naïve, central memory, effector memory and effector on gated CD45.2+ CD3+ CD4+ and CD8+ lymphocytes in control (open symbol) and IFN-α treated (black symbols) animals are indicated and presented as median. (c) Absolute numbers of donor T cell subsets in the lymphoid organs were enumerated following the treatment with rmIFN-α (black symbols) and PBS (open symbols). A nonparametric Mann-Whitney test was performed for comparisons between groups. Data from two representative experiments out of four, including an average of 4 mice per group, are presented. (PDF) [file ppat.1003976.s007.pdf]
