# Supplementary material for: Chronic Exposure to Type-I IFN under Lymphopenic Conditions Alters CD4 T Cell Homeostasis
Source: PLoS Pathog. 2014 Mar 6;10(3):e1003976. doi: 10.1371/journal.ppat.1003976 (PMC3946368; doi:10.1371/journal.ppat.1003976)
Supplement: Table S1 — Viral Infection history from HIV-infected patients described in Figure 1 and Figure 4 . (PDF) [file ppat.1003976.s008.pdf]

Table S1. Viral infection history

				Coinfection history								
**Patient Number**	**Experiment**	**Visit Date Sample tested**	**Months viral load <50 copies/ml**	**Cytomegalovirus (CMV)**	**Epstein–Barr virus (EBV)**	**Hepatitis B virus (HBV)**	**Hepatitis C virus (HCV)**	**Herpes simplex virus (HSV)**	**Influenza A**	**Rotavirus**	**Respiratory syncytial virus (RSV)**	**Varicella zoster virus (VZV)**
Figure 1 - Pt 1.a	Longitudinal Study	4/11/06	-						Isolate parainfluenza virus type 1 (7/14/09), isolate influenza virus type A (1/29/08, 12/11/03) EIA + (1/29/08, 12/11/03)			
Figure 1 - Pt 1.b	Longitudinal Study	7/14/09	28									
Figure 1 - Pt 2.a	Longitudinal Study	7/21/05	-					PCR + (2/16/11)				
Figure 1 - Pt 2.b	Longitudinal Study	10/16/08	8									
Figure 1 - Pt 3.a	Longitudinal Study	3/24/05	-									
Figure 1 - Pt 3.b	Longitudinal Study	1/16/08	20									
Figure 1 - Pt 4.a	Longitudinal Study	12/19/06	-		Low + (5/6/10, 11/27/07, 3/22/07, 1/25/07, 12/26/06), + (1/14/10, 6/18/09, 1/11/07), ND (9/24/09, 2/12/09, 6/5/07)							
Figure 1 - Pt 4.b	Longitudinal Study	3/27/08	11									
Figure 1 - Pt 5.a	Longitudinal Study	12/27/07	-				PCR + (12/27/07), RNA Quantitative: 1571400 copies/mL (6/2/09), 1625400 copies/mL (4/23/09), 8154000 copies/mL (3/3/09), RNA IU: 582,000 IU/mL (6/2/09), 602,000 IU/mL (4/23/09), 3,020,000 (3/3/09)					
Figure 1 - Pt 5.b	Longitudinal Study	9/1/09	16									
Figure 1 - Pt 6.a	Longitudinal Study	2/19/02	-						Isolate- Haemophilus influenzae, heavy (11/12/08)			
Figure 1 - Pt 6.b	Longitudinal Study	5/6/03	11									
Figure 1 - Pt 7.a	Longitudinal Study	8/4/98	-									
Figure 1 - Pt 7.b	Longitudinal Study	12/8/99	13									

				Coinfection history								
**Patient Number**	**Experiment**	**Visit Date Sample tested**	**Months viral load <50 copies/ml**	**Cytomegalovirus (CMV)**	**Epstein–Barr virus (EBV)**	**Hepatitis B virus (HBV)**	**Hepatitis C virus (HCV)**	**Herpes simplex virus (HSV)**	**Influenza A**	**Rotavirus**	**Respiratory syncytial virus (RSV)**	**Varicella zoster virus (VZV)**
Figure 1 - Pt 8.a	Longitudinal Study	2/12/08	-				PCR + (12/23/04, 9/26/02) RNA Quantitative: 5,481,000 copies/mL (6/17/05), 6,075,000 copies/mL (3/11/05) 1,580,000 IU/mL (12/23/04) 455,000 IU/mL (1/30/03), 2,380,000 IU/mL (12/19/02), 4,470,000 IU/mL (10/30/02), 487,000 copies/mL (8/21/02), 4,520,000 copies/mL (8/8/02), RNA IU: 2,030,000 IU/mL (6/17/05), 2,250,000 (3/11/05), 4,266,000 copies/mL (12/23/04), 1,137,500 copies/mL (1/30/03), 5,950,000 copies/mL (12/19/02), 10,012,800 copies/mL (10/30/02), 1,129,840 copies/mL (8/21/02), 8,136,000 copies/mL (8/8/02)					
Figure 1 - Pt 8.b	Longitudinal Study	10/27/09	11									
Figure 1 - Pt 9.a	Longitudinal Study	3/16/04	-									
Figure 1 - Pt 9.b	Longitudinal Study	6/19/08	6									
Figure 1 - Pt 10.a	Longitudinal Study	3/2/01	-									
Figure 1 - Pt 10.b	Longitudinal Study	12/10/03	9									
Figure 4 - Pt 1	Cross-sectional Study	5/29/08	6			HBe Ag: Negative (1/29/07), Positive (3/1/07, 7/18/06, 10/4/05, 4/22/04, 1/20/04), HBs Ag: Repeat Reactive, confirmed positive (4/17/08 1/29/07, 7/18/06, 1/20/04, 7/1/02), DNA quantitative: <116.4 (9/11/12), 58 (11/19/2009, None detected (4/17/2008, 3/1/07, 1/29/07, 7/18/2006, 10/4/05, 4/22/04), DNA IU: <20 (9/11/12), None detected (3/15/11)<10 (11/19/09)						
Figure 4 - Pt 2	Cross-sectional Study	11/19/09	98			DNA Quantitative: 58 copies/ml (11/19/09); DNA-IU: <10 IU/ml (11/19/09)						

				Coinfection history								
**Patient Number**	**Experiment**	**Visit Date Sample tested**	**Months viral load <50 copies/ml**	**Cytomegalovirus (CMV)**	**Epstein–Barr virus (EBV)**	**Hepatitis B virus (HBV)**	**Hepatitis C virus (HCV)**	**Herpes simplex virus (HSV)**	**Influenza A**	**Rotavirus**	**Respiratory syncytial virus (RSV)**	**Varicella zoster virus (VZV)**
Figure 4 - Pt 3	Cross-sectional Study	4/3/03	22									
Figure 4 - Pt 4	Cross-sectional Study	10/9/08	9									
Figure 4 - Pt 5	Cross-sectional Study	11/5/09	33									
Figure 4 - Pt 6	Cross-sectional Study	1/15/09	58									
Figure 4 - Pt 7	Cross-sectional Study	5/22/07	62									
Figure 4 - Pt 8	Cross-sectional Study	4/8/10	28									
Figure 4 - Pt 9	Cross-sectional Study	1/6/05	6									
Figure 4 - Pt 10	Cross-sectional Study	3/1/07	22									
Figure 4 - Pt 11	Cross-sectional Study	12/17/01	27+									
Figure 4 - Pt 12	Cross-sectional Study	12/7/10	7									
Figure 4 - Pt 13	Cross-sectional Study	10/9/08	10									
Figure 4 - Pt 14	Cross-sectional Study	6/16/09	34						Isolate influenza type A 3/13/06			
Figure 4 - Pt 15	Cross-sectional Study	12/8/09	39									
Figure 4 - Pt 16	Cross-sectional Study	8/30/05	9						Isolate- Haemophilus influenzae, heavy (11/12/08)			
Figure 4 - Pt 17	Cross-sectional Study	1/25/11	42					Isolated HSV 11/17/03 from skin lesion				
Figure 4 - Pt 18	Cross-sectional Study	4/17/07	13									
Figure 4 - Pt 19	Cross-sectional Study	11/5/09	72									
Figure 4 - Pt 20	Cross-sectional Study	11/1/07	19									
Figure 4 - Pt 21	Cross-sectional Study	9/9/08	52									
Figure 4 - Pt 22	Cross-sectional Study	40561	10									
Figure 4 - Pt 23	Cross-sectional Study	40463	19									
Figure 4 - Pt 24	Cross-sectional Study	3/1/11	47									
Figure 4 - Pt 25	Cross-sectional Study	8/10/01	12									
Figure 4 - Pt 26	Cross-sectional Study	9/11/08	15		PCR +(5/18/10, 2/9/10, 10/2/09, 6/12/09, 4/13/09, 2/26/09, 11/06/08, 7/17/08, 3/14/08, 1/3/08, 9/27/07, 7/24/07, 6/21/07, 5/23/07, 4/23/07) Low + (9/20/10, 4/17/07), - 12/09/10, ND 5/8/07							PCR + (5/23/07, 4/23/07, 4/12/07), PCR - (4/17/07, 6/4/07)
Figure 4 - Pt 27	Cross-sectional Study	4/11/00	33									

				Coinfection history								
**Patient Number**	**Experiment**	**Visit Date Sample tested**	**Months viral load <50 copies/ml**	**Cytomegalovirus (CMV)**	**Epstein–Barr virus (EBV)**	**Hepatitis B virus (HBV)**	**Hepatitis C virus (HCV)**	**Herpes simplex virus (HSV)**	**Influenza A**	**Rotavirus**	**Respiratory syncytial virus (RSV)**	**Varicella zoster virus (VZV)**
Figure 4 - Pt 28	Cross-sectional Study	2/19/08	13		PCR + (2/15/2006, 1/27/06), Negative 4/28/05							
Figure 4 - Pt 29	Cross-sectional Study	8/5/08	17						Isolate parainfluenza type 1 (7/14/09), EIA + (1/29/08)			
Figure 4 - Pt 30	Cross-sectional Study	1/21/10	41									
Figure 4 - Pt 31	Cross-sectional Study	3/22/11	37					PCR + (02/16/11)				
Figure 4 - Pt 32	Cross-sectional Study	9/21/10	8		7447550 copies/mL (6/15/13), 850 copies/mL (8/15/2012), PCR + (10/22/2009)							
Figure 4 - Pt 33	Cross-sectional Study	12/7/10	19									
Figure 4 - Pt 34	Cross-sectional Study	6/22/06	7		PCR + (3/15/06, 11/3/05), PCR - (7/20/05)			Culture + (03/22/06)				
Figure 4 - Pt 35	Cross-sectional Study	4/23/03	17				PCR + (1/4/07), RNA IU: 24900000 IU/mL (10/5/11), 13300000 IU/mL (6/14/11), 64,300 IU/mL (11/4/08), 257000 IU/mL(1/4/07) RNA Quantitative: 67230000 copies/mL (10/5/11), 35910000 copies/mL (6/14/11), 173610 copies/mL (11/4/08), 693900 copies/mL (1/4/07)					
Figure 4 - Pt 36	Cross-sectional Study	5/14/03	6						Isolate Influenza A 12/26/03			
Figure 4 - Pt 37	Cross-sectional Study	10/13/09	9									
Figure 4 - Pt 38	Cross-sectional Study	2/8/05	7			HBs Ag: Reactive (02/08/05)		Culture + (7/20/04)				
Figure 4 - Pt 39	Cross-sectional Study	4/28/09	12									
Figure 4 - Pt 40	Cross-sectional Study	12/16/04	8		PCR + (7/22/04), ND (8/18/09)							
Figure 4 - Pt 41	Cross-sectional Study	12/18/07	6	PCR Low + (10/23/08, 6/5/07, 5/10/07, 4/11/07, 3/29/07), PCR - (7/1/08, 7/3/07, 2/11/09) PCR + (4/25/07)	PCR + (1/13/10), PCR Low + (11/12/09, 6/3/09, 2/11/09, 7/1/08, 3/13/08, 12/18/07), ND (10/23/08, 9/25/07)							PCR + (7/1/08), PCR - 4/4/07

				Coinfection history								
**Patient Number**	**Experiment**	**Visit Date Sample tested**	**Months viral load <50 copies/ml**	**Cytomegalovirus (CMV)**	**Epstein–Barr virus (EBV)**	**Hepatitis B virus (HBV)**	**Hepatitis C virus (HCV)**	**Herpes simplex virus (HSV)**	**Influenza A**	**Rotavirus**	**Respiratory syncytial virus (RSV)**	**Varicella zoster virus (VZV)**
Figure 4 - Pt 42	Cross-sectional Study	9/29/03	8			HBs Ag: + (1/25/11, 11/6/09), Repeat Reactive (11/8/05) Hbe Ag: Reactive (Negative) (3/26/13), + (9/8/09, 6/6/06, 11/8/05, 8/18/04) DNA Quantitative: <116.4 copies/mL (3/6/12), 338 copies/mL (9/22/09), 588 copies/mL (9/08/09), 1996 copies/mL (9/01/09), 6577 copies/mL (8/18/09), 7217 copies/mL (6/8/09), 4842 copies/mL (12/16/08), 21243 copies/mL (5/27/08), 4655 copies/mL (7/6/06), 6102 copies/mL (6/6/06), 10941 copies/mL (4/13/06), 500 copies/mL (11/8/05), 92600 copies/mL (8/18/04), DNA IU: <20 IU/mL (3/6/12), 58 IU/mL (9/22/09), 101 IU/mL (9/8/11), 343 IU/mL (9/1/09), 1130 (8/18/09), 1240 IU/mL (6/8/09), 832 IU/mL (12/16/08), 3650 IU/mL (5/27/08), 885 IU/mL (7/6/06), 1160 IU/mL (6/6/06), 2080 IU/mL (4/13/06), <95 IU/mL (11/8/05)						
Figure 4 - Pt 43	Cross-sectional Study	7/29/04	12									

Patient Number	Experiment	Visit Date Sample tested	Months viral load <50 copies/ml	Coinfection history								
				Cytomegalovirus (CMV)	Epstein–Barr virus (EBV)	Hepatitis B virus (HBV)	Hepatitis C virus (HCV)	Herpes simplex virus (HSV)	Influenza A	Rotavirus	Respiratory syncytial virus (RSV)	Varicella zoster virus (VZV)
Figure 4 - Pt 44	Cross-sectional Study	11/8/05	16			HBs Ag: Reactive (1/13/09), Repeat reactive (8/16/05, 9/3/04, 2/17/04), Hbe Ag: Reactive 9/6/02, DNA Quantitative: None detected (6/23/05), 927 copies/mL (9/3/04), 37,600,000 diluted 1:1000 copies/mL (2/17/04), 2,500,000,000 copies/mL dilute 1:10,000 (11/24/03), >500,000 copies/mL (9/6/02), 322,000 copies/mL (6/26/02)		Culture + (10/15/04)				
Figure 4 - Pt 45	Cross-sectional Study	5/5/09	17		PCR + (3/11/10)							
Figure 4 - Pt 46	Cross-sectional Study	11/13/08	12									
Figure 4 - Pt 47	Cross-sectional Study	2/24/10	11									
Figure 4 - Pt 48	Cross-sectional Study	12/18/08	36		PCR + (3/15/06, 11/3/05, 7/20/05)			Culture + (3/22/06, 3/15/06)				
Figure 4 - Pt 49	Cross-sectional Study	7/20/10	40				RNA Quantitative: 143640 copies/mL (10/18/12), 10,530,000 copies/mL (8/23/05), <1,620 copies/mL (6/7/05) None detected (5/3/09), RNA IU: 53200 IU/mL (10/18/12), 3,900,000 IU/mL, <600 IU/mL (6/7/05), none detected (5/3/09)	PCR + (10/2/12)				
Figure 4 - Pt 50	Cross-sectional Study	3/29/10	6					Culture + (7/24/07)				
Figure 4 - Pt 51	Cross-sectional Study	4/28/09	8									
Figure 4 - Pt 52	Cross-sectional Study	12/3/10	21		PCR Low + (12/4/08)							
Figure 4 - Pt 53	Cross-sectional Study	3/29/11	113			HBs Ag: + (1/5/10, 7/2/09), Repeat reactive known positive (4/6/04, 2/11/03), DNA Quantitative: None detected (9/24/13, 5/4/10, 2/26/08, 6/26/03, 10/3/02), <169 copies/mL (3/29/11), 58 copies/mL (1/5/10), DNA IU: <29 IU/mL (3/29/11), <10 IU/mL (1/5/10)						
